# Salidroside reduces neuropathology in Alzheimer’s disease models by targeting NRF2/SIRT3 pathway

**DOI:** 10.1186/s13578-022-00918-z

**Published:** 2022-11-04

**Authors:** Yuyuan Yao, Zhichu Ren, Ruihan Yang, Yilan Mei, Yuying Dai, Qian Cheng, Chong Xu, Xiaogang Xu, Sanying Wang, Kyoung Mi Kim, Ji Heon Noh, Jian Zhu, Ningwei Zhao, Yong U. Liu, Genxiang Mao, Jian Sima

**Affiliations:** 1 Laboratory of Aging Neuroscience and Neuropharmacology, School of Basic Medicine and Clinical Pharmacy, China Pharmaceutical University, Nanjing, 210009 China; 2 Zhejiang Provincial Key Lab of Geriatrics and Geriatrics, Institute of Zhejiang Province, Department of Geriatrics, Zhejiang Hospital, Hangzhou, 310030 China; 3 Department of Biological Sciences, Chungnam National University, Daejeon, 34134 Korea; 4 Department of Biochemistry, Chungnam National University, Daejeon, 34134 Korea; 5 Department of Psychology, Eastern Illinois University, Charleston, IL 61920 USA; 6 China Exposomics Institute, 781 Cai Lun Road, Shanghai, 200120 China; 7 Laboratory for Neuroscience in Health and Disease, Guangzhou First People’s Hospital, South China University of Technology, Guangzhou, 510180 China

**Keywords:** Alzheimer’s disease, Mitochondrial protection, SAL, SIRT3, NRF2

## Abstract

**Background:**

Neurite dystrophy is a pathologic hallmark of Alzheimer’s disease (AD). However, drug discovery targeting neurite protection in AD remains largely unexplored.

**Methods:**

Aβ-induced neurite and mitochondrial damage assays were used to evaluate Aβ toxicity and the neuroprotective efficacy of a natural compound salidroside (SAL). The 5×FAD transgenic mouse model of AD was used to study the neuroprotective function of SAL. To verify the direct target of SAL, we used surface plasmon resonance and cellular thermal shift assays to analyze the drug-protein interaction.

**Results:**

SAL ameliorates Aβ-mediated neurite damage in cell culture. We further reveal that SAL represses mitochondrial damage in neurites by promoting mitophagy and maintaining mitochondrial homeostasis, dependent on an NAD-dependent deacetylase SIRT3. In AD mice, SAL protects neurite morphology, mitigates Aβ pathology, and improves cognitive function, which are all SIRT3-dependent. Notably, SAL directly binds to transcription factor NRF2, inhibits its degradation by blocking its interaction with KEAP1 ubiquitin ligase, and then advances NRF2-mediated SIRT3 transcription.

**Conclusions:**

Overall, we demonstrate that SAL, a potential anti-aging drug candidate, attenuates AD pathology by targeting NRF2/SIRT3 pathway for mitochondrial and neurite protection. Drug discovery strategies focusing on SAL may thus provide promising therapeutics for AD.

**Supplementary Information:**

The online version contains supplementary material available at 10.1186/s13578-022-00918-z.

## Background

The major neuropathological features of Alzheimer’s disease (AD) are the presence of beta-amyloid (Aβ) plaques, neurofibrillary tangles, and neuronal loss in brain, firstly reported by Dr. Alois Alzheimer in his original case of the disease that would eventually bear his name [1]. Abnormal neuronal processes surrounding Aβ plaques, known as dystrophic neurites (DNs), are now believed to be another critical hallmark of AD, specially at early stages [2, 3]. Interestingly, in the early twentieth century, the famous neuroscientist Ramón y Cajal already provided a hypothesis about the development of DNs in AD [4]. Recent evidence has shown that DNs appear to surround and enter adjacent Aβ plaques, which may disrupt microtubes, impair axonal transport and lead to exacerbation of Aβ pathology [5, 6].

Aβ released from senile plaques, is toxic to nearby neurites including axons or dendrites or some combination of both. The mechanisms of Aβ toxicity involve inflammation, membrane perforation, synaptic failure, oxidative stress, and mitochondrial dysfunction [7, 8]. Among these mechanisms, much attention has been focused on mitochondria, as a central hub, regulating stress sensing, energy metabolism and neuronal death. Notably, mitochondria are recently shown to be vital for modulating neurite retraction [9], which can be induced by both extracellular fibrillar Aβ and intracellular aggregated Tau in AD at early stages. In fact, targeting mitochondria has emerged as a promising approach for neuroprotection. In the past decade, increasing drug candidates that target “in and out” the mitochondria have been investigated from preclinical stages to clinical trials in neurodegenerative diseases (NDs) [10, 11].

Sirtuins (SIRTs) are a family of proteins that act as nicotinamide adenine dinucleotide (NAD^+^) dependent deacetylases. In mammals, seven SIRTs exist, including three members, SIRT3, 4, and 5, that localize exclusively within the mitochondria. Mounting evidence has shown that SIRTs, particularly SIRT1 and SIRT2, target multiple substrates such as PGC-1α, NF-κB, FOXO1/3, and superoxide dismutase 2 (SOD2), and play a protective function against AD pathogenesis [12, 13]. In addition, mitochondrial SIRT3-5 may also function as anti-aging or neuronal/synaptic protective agents [14–16], but their possible activities against NDs needs to be further validated. In the recent few years, mitophagy, an autophagy pathway by removing damaged mitochondria, is proved to be compromised in AD, and promotion of mitophagy improves AD pathologies [17, 18]. In this respect, pharmacological rescue of impaired mitophagy is now considered to be beneficial for AD therapeutics [19, 20]. SIRT3 is known to regulate mitochondrial function including mitophagy in nervous system [21], but the precise interplay between SIRT3 dynamics and mitophagy process in AD is still unclear. It will be also interesting to determine the SIRT3 function on other aspects of mitochondrial biology, including mitochondrial morphology, mitochondrial membrane potential (MMP), and reactive oxygen species (ROS) production.

Salidroside (SAL) is a glucoside of tyrosol identified in the plant *Rhodiola rosea*. As a natural bioactive compound, it has been studied as a potential neuroprotective molecule for the treatment of many neurological disorders including AD [22]. In fact, SAL administration improves learning and memory in various animal models, but the underlying mechanisms are vague, possibly by modulation of neuroinflammation, oxidative stress, and cell death [22]. It is important to note that no direct targets of SAL have so far been revealed, which explains the uncategorized mechanism of action and hinders its clinical application. In AD pathogenesis, neurite dystrophy is thought to be an earlier event before neuronal death. SAL has shown protective efficacy on hippocampal neuronal death [23], but whether it acts in the homeostatic maintenance of neurites or the organelles inside remains unknown.

Here, we report that SAL can prevent Aβ-induced neuritic toxicity in cell culture. The protective function of SAL is mediated by SIRT3-dependent regulation of mitochondrial homeostasis and mitophagy. In the 5×FAD mouse model of AD, SAL treatment protects neurite morphology, reduces Aβ pathology, and improves cognitive function, which are dependent on SIRT3 as well. Strikingly, we find that SAL directly binds to a transcription factor NRF2 and inhibits its degradation by disrupting the formation of NRF2-KEAP1 protein complex, then upregulates NRF2-mediated SIRT3 transcription. Thus, our findings reveal a novel function of SAL for neurite protection in AD by directly targeting NRF2/SIRT3 pathway, which may also represent a general mechanism of SAL protection in other NDs.

## Materials and methods

### Mice

C57BL/6, 5×FAD (JAX, Stock # 034848), and *Sirt3*^*flox*^ (JAX, Stock # 031201) mice were used in this study. *Sirt3*^*flox*^ mice were used for primary neuronal culture. Mice were raised in the temperature of 25 ± 2 °C, relative humidity 70% and in a 12 h light/dark room at with free access to food and water. For in vivo animal study as described in Fig. 5A, 16 weeks (w) old WT or 5×FAD mice (male:female ≈ 1:1) were randomly divided into 4 groups with or without AAV injection. After 1 w, 2 groups of mice were orally administrated SAL until 29 w old for behavioral tests, then for the following histological experiments at 30 w old. For SAL administration, we used a previously described procedure [24]. In brief, 0.3 mg/mL SAL was dissolved in drink water in 50 mL bottle with free access and changed once daily. We calculated the amount of drink water based on ~ 3–5 mL/mouse/day and found no body weight change during the procedure. All experiments were conducted in accordance with the regulations for the Administration of Affairs Concerning Experimental Animals and approved by the China Pharmaceutical University Animal Ethics Committee.

### Cell culture

SH-SY5Y cells were cultured in DMEM with 10% FBS. The neuronal differentiation of SH-SY5Y was conducted as previously described [25], with modifications. In brief, cells were maintained in 10% FBS DMEM. One day after plating on 50 mg/mL Poly-d-lysine (PDL) (Beyotime) coated 35 mm dishes, changed the media to 1% FBS MEM plus with 10 μM ATRA (MCE) and cultured for 3 days, then changed to Neurobasal medium (Thermo) plus with B27 supplement (Thermo) and cultured for another 6 days.

Primary neuronal culture was performed as described [26]. In brief, cortical neurons were isolated from brains of P0 homozygote *Sirt3*^*floxp*^ mice and cultured in Neurobasal medium plus with B27 supplement in 6-well plates coated with PDL. In some conditions, neurons are transduced with AAV particles expressing each indicated proteins (Additional file 1: Fig. S5) at day 3. In neurite morphology analysis, differentiated SH-SY5Y or primary neurons were treated with PBS, or 50 μM of SAL (Provided by Dr. Gengxiang Mao) for 24 h, followed by adding 10 μM of Aβ oligomers, or 25 μM of CCCP (MCE) for 72 h, and then used for further experiments. In mitochondrial morphology observation, Aβ or CCCP treatment was reduces to 48 h or 5 h, respectively.

### Preparation of Aβ oligomers

Aβ42 peptide was purchased from GenScript. The oligomerization of Aβ42 was conducted as described [27]. In short, 1 mM Aβ42 peptides were dissolved in hexafluoroisopropanol (HFIP) to remove any pre-existing aggregates and β-sheet secondary structure. After 30 min incubation at RT, aliquots of Aβ42 were transferred to SpeedVac and dry for 1 h to evaporate HFIP, until a clear peptide film was observed and then stored at − 80 °C. Prior to use, Aβ42 film were dissolved with DMSO at the concentration of 5 mM, vortexed 30 s and sonicated for 10 min in a water bath, incubated at 4 °C for another 24 h for the oligomerization.

### MMP analysis, ROS assay and mitochondria isolation

The mitochondrial membrane potential (MMP) was measured using a JC-1 assay kit (Beyotime) by following the manufacturer’s protocol. Images were captured by a fluorescence microscope (Olympus, IX73). The ratio of red (aggregates) to green (monomers) fluorescent intensity was measured using ImageJ 1.53 software.

The ROS assays were performed using the fluorescent probe DCFH (MCE). Briefly, cells are washed and incubated with PBS containing 10 μM DCFH for 20 min, then quickly washed with PBS twice for observation under fluorescence microscope.

Mitochondria were isolated by following an exactly same protocol described from Abcam website (https://docs.abcam.com/pdf/protocols/mitochondrial-purification-protocol-for-western-blot-samples.pdf).

### Immunofluorescence (IF), immunohistochemistry (IHC) and mKeima assay

For IF, cells were fixed in 4% PFA and followed a protocol reported in our previous paper [26]. For IHC, mice were sacrificed and brain extracted for cryostat sectioning. 30 μm frozen slices were sectioned by Leica CM1950 microtome and IHC was conducted using our protocol described before [26]. Antibodies used in this study were listed in Additional file 1: Table S1.

An mKeima-Red-Mito-7 plasmid (Addgene) was transfected into SH-SY5Y cells to generate a stable cell line expressing mKeima localized on mitochondria. A confocal microscope (Olympus, FV3000) was used to detect two wavelength setting including a green channel (λEx = 440 nm, λEm = 610–710 nm) and a red channel (λEx = 589 nm, λEm = 610–710 nm). The mitophagy index (ratio of red signal to green) was quantified using ImageJ 1.53 software.

### Immunoblotting, immunoprecipitation (IP) and qPCR

Immunoblotting, IP and qPCR were performed using our standard protocols [28]. For protein extraction from mouse hippocampal tissues, mice were sacrificed and perfused with saline, and hippocampi were isolated and lysed using a kit (Genuin Biotech) following the manufacture’s protocol. All primers used for qPCR were listed in Additional file 1: Table S2.

### Preparation of adeno-associated virus (AAV), lentivirus and adenovirus

The original vector pAAV-CAG-GFP (Addgene) was reconstructed for our experiments. In brief, the GFP was replaced by MitoGFP, then a T2A-NLS-Cre was added following MitoGFP. For knockdown of *Sirt3*, the shRNA sequence targeting mouse *Sirt3* (5′-GCCCAATGTCACTCACTACTT-3′) were cloned into the vector pAAV-EGFP-U6-shRNA (Addgene). AAV production and purification were conducted by following the same protocol from Addgene website (https://www.addgene.org/protocols/aav-production-hek293-cells/; https://www.addgene.org/protocols/aav-purification-iodixanol-gradient-ultracentrifugation/). We used the protocols established in our laboratory for the production of lentivirus and adenovirus [26]. The shRNA sequence targeting human SIRT3 was: 5′-GCCCAACGTCACTCACTACTT-3′. The structures of viral vectors used in this study were listed in Additional file 1: Fig. S5.

### Brain stereotaxic injection, Morris water maze and Y-maze

Brain stereotaxic injection was performed using our protocol described before [26]. For hippocampal SIRT3 KD, 2 μL of AAV particles were bilaterally injected into the hippocampal CA1 region at − 2.2 mm from bregma, mediolateral 1.7 mm, depth 2.4 mm of mouse brains. All mice were placed on a heating pad until they recovered from the surgery. Morris water maze was done using a same protocol we described previously [26]. Y-Maze experiments were performed as described [29].

### Golgi staining

Golgi staining were performed based on a protocol previously described [30], with modification to be compatible with cryosectioning. In short, mouse is sacrificed and perfused with saline, and whole brains were isolated and fixed in 4% PFA for 36–48 h. After fixation, brain tissues were soaked in 30 mL of 3% potassium dichromate for a week and the solution was replaced daily. Next, tissue blocks were washed in increasing concentrations (0.25, 0.50, 0.75%) of silver nitrate for 5 min each, then placed in 2% silver nitrate in the dark for another 7 days. After that, tissued were transferred into the cryoprotectant solution that contains 20% sucrose and 15% glycerol at 4 °C for 48 h, then embedded in OCT medium (Leica) and stored in − 80 °C. 100 μm-thick sections were cut by a cryostat microtome (Leica), and dehydrated in each 50%, 75%, 95%, and 100% (twice) of ethanol for 5 min respectively, then finally transferred to xylene for 5 min. Slices were mounted and then observed under a microscope (Olympus, IX73).

### Surface plasmon resonance (SPR) and cellular thermal shift assay (CETSA)

NRF2 protein was expressed and purified by a prokaryotic expression combined with immobilized metal affinity chromatography (IMAC) system. In brief, human NRF2 cDNA was cloned into an expression vector pET-24a (+) and expressed with a fused 6× his tag in BL21 (DE3) cells (Sangon Biotech), then purified using a kit (Beyotime) following the manufacture’s protocol. SPR assays were performed using the Biacore T200 by following the manufacture’s operating manual.

CETSA experiments were carried out using a protocol described before [31]. In short, SH-SY5Y cells were cultured and treated with or without 500 μM SAL for 4 h. Cells were washed twice with PBS, and resuspend in PBS containing protease inhibitors and aliquoted 100 μL with 3 × 10^6^ cell per tube. Each tube containing cells was incubated the at setting temperature (45–65 °C) for 3 min, at 25 °C for 3 min, and snap-freezed in liquid nitrogen for 2 min, and thawed at 25 °C for 2 min. Repeat the snap-freeze thaw process for 2 more times. Centrifuge the cell lysate at 20,000 rpm for 20 min and collected supernatant for immunoblotting.

### Morphological analysis of neurites, mitochondria and dendritic spines

For neurite analysis, brightfield or fluorescent images were collected by Olympus IX73 microscope. Each image consists of 20–30 cells and total neurite length and cell numbers was measured using ImageJ software, then the average total neurite length per cell was calculated. At least 7 random images from 3 different experiments were used for quantification. For mitochondrial morphology investigation, a 60× objective (oil, NA1.4) was used to collect images. Each cell/neurite contains more than 20 clear mitochondrial segments. The length or density of mitochondria in neurites were measured and calculated using ImageJ software. At least 15 mitochondrial segments in each neurite and more than 20 neurites (total number (n) of mitochondrial segments, n ≥ 300). Images of Golgi-stained neurons were collected by a 100× objective (oil, 1.4) and analyzed by ImageJ software. Each image contains more than 5 clear single neurites and more than 12 images (n ≥ 60) from at least 6 mice were used for quantification.

### Data and statistical analyses

All data showed as mean ± SD, unless otherwise specified. One-way ANOVA followed by Tukey’s multiple comparisons test, unless otherwise specified, was used for measure group differences in multiple groups. GraphPad Prism 8.0 was used for the statistical analysis. *P* values < 0.05 was considered statistically significant.

## Results

### SAL alleviates neurite and mitochondrial injury induced by toxic CCCP or Aβ42 oligomers

**Fig. 1:**
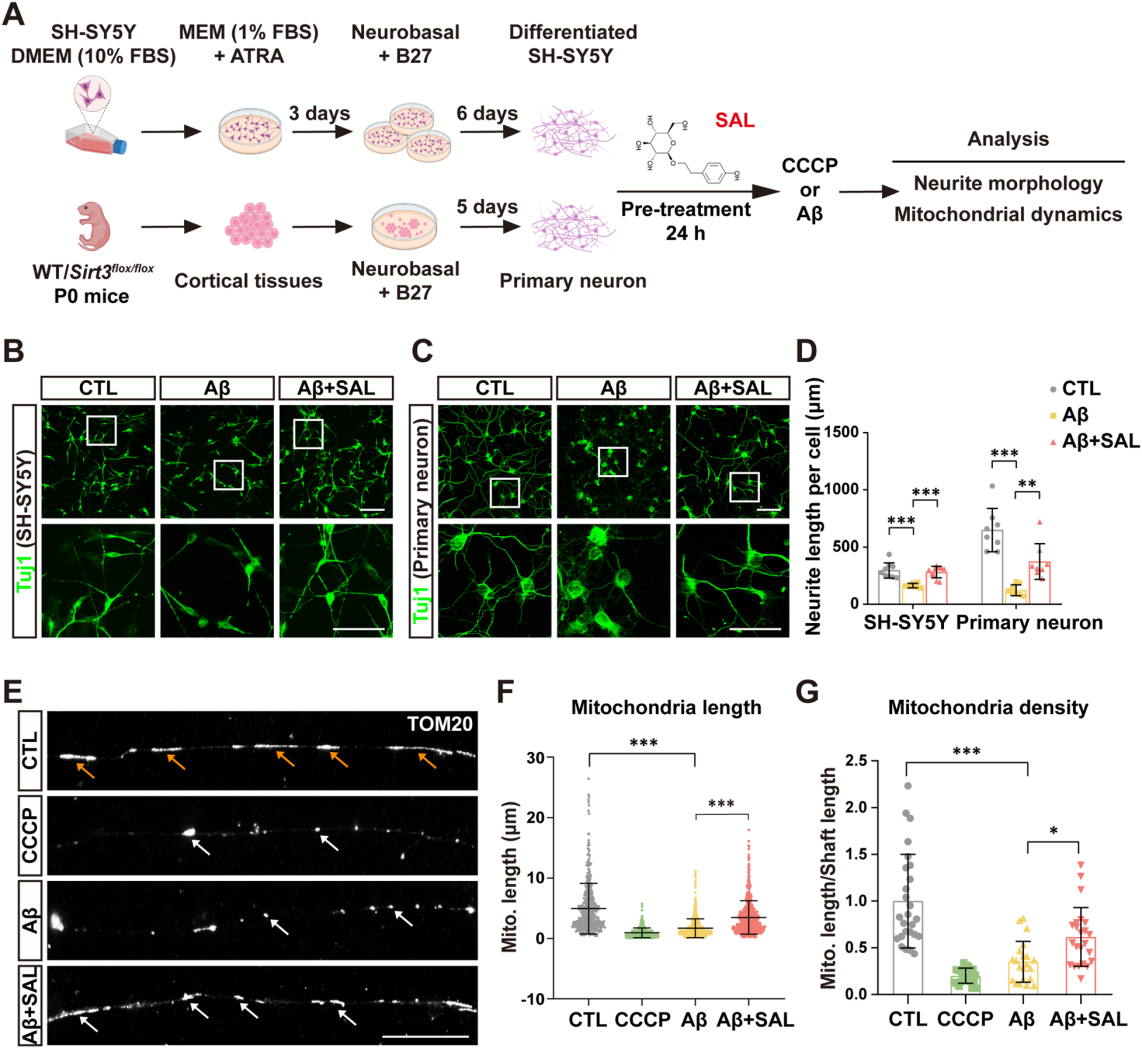
SAL ameliorates Aβ-induced neurite and mitochondrial damage. **A** Schematic overview shows the workflow of SH-SY5Y differentiation and primary neuronal culture for SAL efficacy study by assessing neurite morphology and mitochondrial dynamics. **B** Immunofluorescence (IF) of neuronal marker Tuj1 (green) in differentiated SH-SY5Y cells, treated with indicated Aβ42 oligomers or SAL (see “Materials and methods”). White rectangles indicate amplified images at lower panels. Scale bar, 50 μm. **C** As in **B**, except primary neurons used. **D** Quantification of neurite length of **B** and **C**. The average neurite length without additional treatment was regarded as control (CTL). In each condition, at least 7 random images (each image includes ≥ 20 cells) from 3 independent experiments were used for quantification. **E** IF of TOM20 shows the mitochondrial segments in differentiated neurites of SH-SY5Y cells with indicated treatments. Scale bar, 10 μm. The quantification shows the length of mitochondrial (mito.) segments (n ≥ 400) in **F**, and the mitochondrial density indicating by the ratio of mito. length to occupied neurite (shaft) length (n ≥ 20) in **G**. Error bars indicate the mean ± SD from at least 20 neurites of 3 independent experiments. *P < 0.05, **P < 0.01, ***P < 0.001; one-way ANOVA test

We hypothesized that SAL might protect neuronal processes (also known as neurites) against damage in AD pathogenesis. To test this hypothesis, we first carried out experiments for neurite differentiation of SH-SY5Y cells using an established protocol (Fig. 1A), and then checked the SAL effect on neurites with or without the stimulation of mitochondrial toxin CCCP or Aβ42 oligomers. Previous findings suggested that 50 μM SAL had no toxic effect and showed good protective efficacy in cultured cells [32, 33]. Our data also showed that 50 μM SAL had better efficacy than 5 μM SAL on neurite extension of SH-SY5Y cells without cell toxicity (Additional file 1: Fig. S1). We thus used 50 μM SAL for the next cell culture studies. The brightfield images of neurite morphology indeed showed a protective action of SAL on neurite length under CCCP stimulation (Additional file 1: Fig. S2A–C). Compared to CCCP, Aβ42 also induced a weaker neurite injury of SH-SY5Y cells shown by fragmented processes, which was also reduced by SAL pre-treatment, as indicated by immunofluorescence (IF) staining of Tuj1 (a neuritic marker) (Fig. 1B, D). Of note, in primary neuronal culture, SAL exerted a remarkable action in neurite protection upon Aβ stimulation, with 2.7-fold increase of the total length, compared to PBS control (Fig. 1C, D). We further found that simultaneous incubation of SAL along with Aβ also inhibited Aβ-induced neurite damage by ~ 27.3% (Additional file 1: Fig. S3).

Neurite injury is highly correlated to the impairment of mitochondria inside the processes. To assess whether SAL prevents mitochondrial damage stimulated by CCCP or Aβ42, we used IF of TOM20 (a mitochondrial marker) to observe their morphology in SH-SY5Y cells. As expected, mitochondria were shortened and fragmented by CCCP stimulation. Again, SAL repressed mitochondrial shortening by ~ 22.1% and their fragmentation by ~ 26.1% in soma (Additional file 1: Fig. S2D–F). We further assessed the mitochondrial protection of SAL on neurites. Like its protective action against CCCP in somata, SAL also remarkably inhibited mitochondrial truncation and fractionalization in neurites induced by Aβ42 (Fig. 1E–G).

These data suggest that SAL ameliorates CCCP or Aβ induced neurite and mitochondrial impairment.

### SAL promotes neuronal mitophagy in both somata and neurites upon Aβ stimulation

**Fig. 2:**
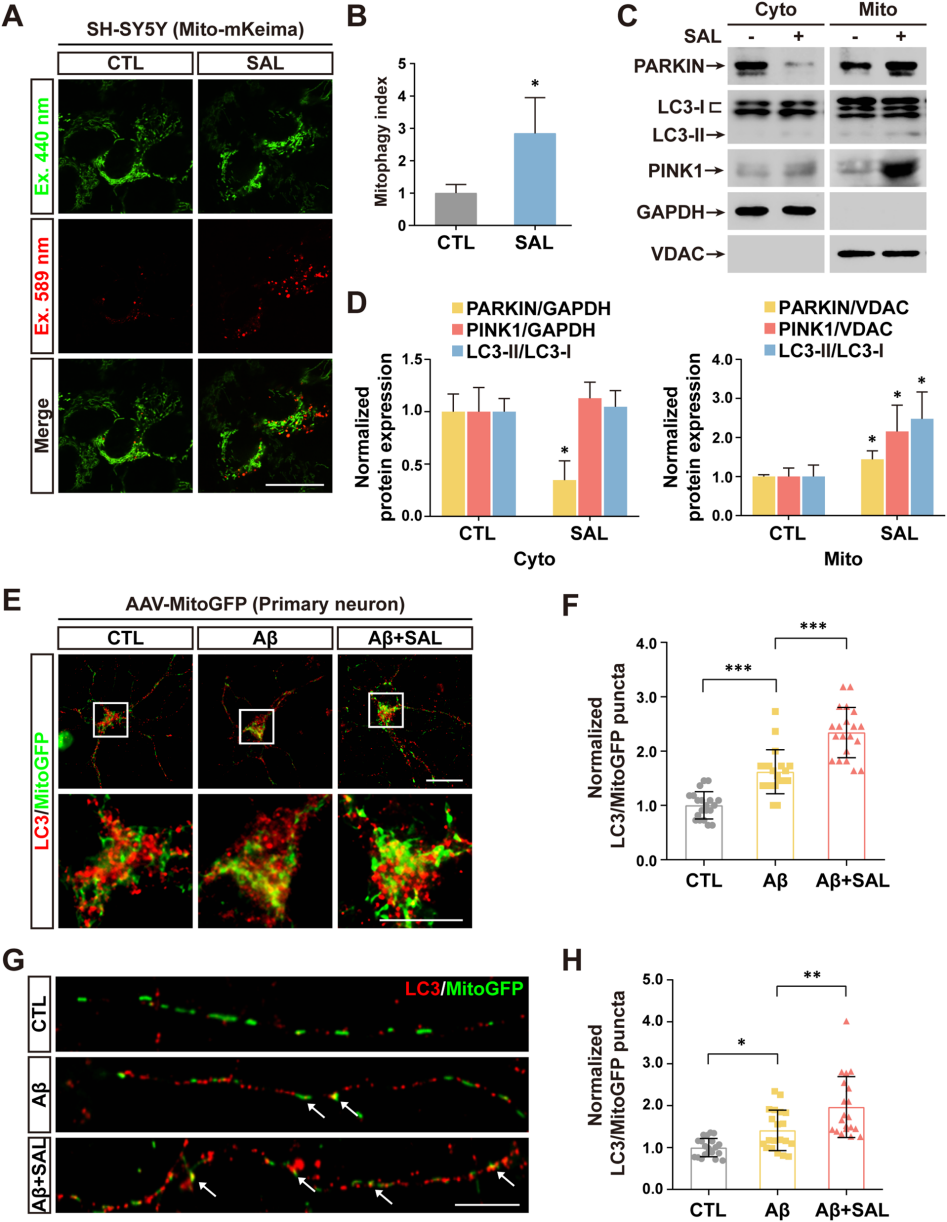
SAL promotes mitophagy in somata and neurites. **A** Images of mito-mKeima expressing SH-SY5Y cells treated with SAL or PBS (CTL). 440 nm excitation (green) labels the healthy mitochondria, whereas 589 nm excitation (red) represents the mitochondria delivered to lysosomes for mitophagy. **B** Quantification of mitophagy index (ratio of fluorescence intensity of λEx = 589 nm against λEx = 440 nm) in **A**. Data from at least 7 random images in each condition from 3 independent experiments. **C** Immunoblotting shows the levels of indicated proteins extracted from cytosol (left panels) and mitochondria (right panels) of SH-SY5Y cells treated with or without SAL. GAPDH and VDAC as cytosolic (Cyto) and mitochondrial (Mito) fraction in **C**. **D** Quantification of band intensity in **C**. Data from 3 independent experiments. **E** IF images of cortical neurons transduced with AAV particles expressing mitoGFP, with indicated treatments. The puncta are labeled with autophagic marker LC3 (red) and mitochondrial GFP (green) and shown in upper panels. Images in white rectangles are amplified and shown in lower panels. **F** Quantitation shows the number of puncta co-labeled with LC3 and MitoGFP. **G**, **H** As described in **E** and **F**, except images of neurites are shown and quantified. Error bars indicates mean ± SD from at least 20 cells/neurites from 3 independent experiments (n ≥ 20). *P < 0.05, **P < 0.01, ***P < 0.001; one-way ANOVA test. Scale bar, 10 μm

Recent evidence refers that activation of mitophagy reverses Aβ pathology in AD mice [19, 34]. These findings lead us to test whether SAL elevates neuronal mitophagy. We first generated a derivative of the SH-SY5Y stable cell line expressing Mito-mKeima, to measure the level of mitophagy. The mKeima assays clearly showed a promotion of mitophagy around 2.8-fold in soma after SAL treatment (Fig. 2A, B). This result was further confirmed by fluorescence images of LC3-GFP and MitoTracker co-localization (Additional file 1: Fig. S4). In addition, we extracted mitochondrial protein from SH-SY5Y cells and examined the markers of mitophagic pathway. Immunoblotting showed that SAL indeed upregulated mitochondrial levels of PARKIN, PINK1 and LC3-II (Fig. 2C, D), suggesting an increase of mitophagy.

By using adeno-associated virus (AAV) particles to express MitoGFP, which specifically labels mitochondria (Additional file 1: Fig. S5A), we transduced primary neurons followed by the treatment with Aβ. IF images of LC3 (red) and MitoGFP (green) co-localization showed that Aβ elevated mitophagy levels, indicated by increased yellow puncta, in somata. Notably, SAL further raised the number of yellow puncta (MitoGFP/LC3 co-localization) by 1.4-fold, compared to Aβ alone, demonstrating the augmentation of mitophagy upon SAL treatment (Fig. 2E, F). To evaluate the mitophagy levels in neurites, we further analyzed the LC3 and MitoGFP co-labeling and found that SAL treatment accelerated mitophagy by 1.5-fold, compared to stimulation with Aβ alone (Fig. 2G, H).

Our data imply that SAL promotes basal mitophagy levels and also exacerbates Aβ-induced mitophagy either in somata or in neurites. Possibly, SAL-triggered elevation of mitophagy may act as a protective mechanism against neurite injury, likely by increasing the clearance of damaged mitochondria [35].

### SAL-activated mitophagy is dependent on SIRT3 expression

**Fig. 3:**
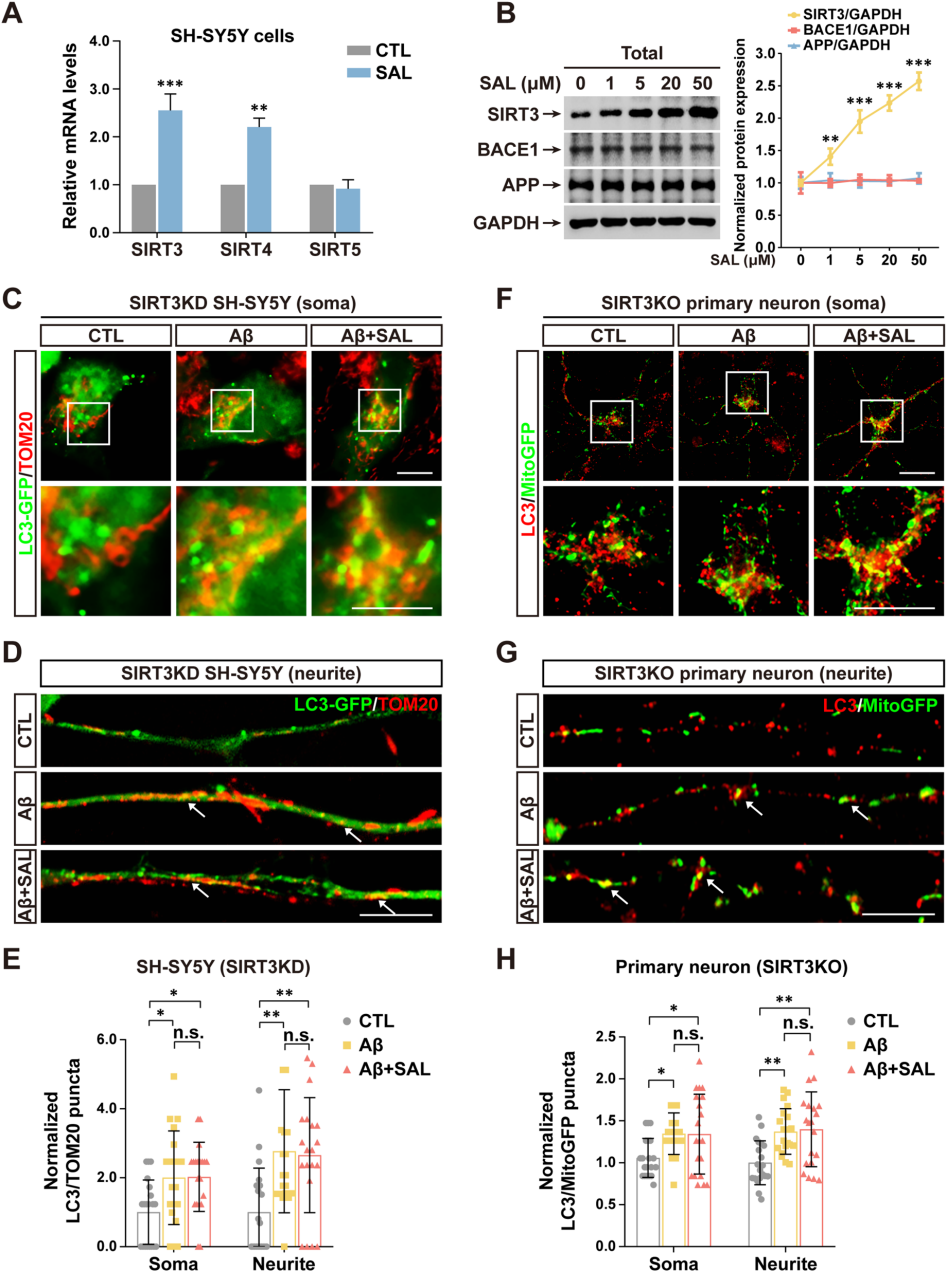
SIRT3 is indispensable for SAL-mediated promotion of mitophagy. **A** qPCR shows the mRNA levels of SIRT3, 4 and 5 in SH-SY5Y cells treated with or without SAL. **B** Immunoblotting shows the levels of indicated proteins in SH-SY5Y cell treated by SAL with each indicated concentration. GAPDH as a loading control. **C** IF shows the colocalization of LC3-GFP and TOM20 in somata of SH-SY5Y cells with SIRT3 KD. Images in rectangles are amplified shown in lower panels. **D** As in **C**, except images of neurites are shown. Arrows indicate co-labeled yellow puncta. **E** Quantification shows the number of puncta co-labeled with LC3 and TOM20 in somata. **F**, **G** As in **C**, **D**, except *SIRT3*^*flox/flox*^ cortical neurons transduced with AAV particles encoding mitoGFP and Cre were used and indicated as SIRT3 KO (see Additional file 1: Fig. S5). **H** As in **E**, except SIRT3 KO cortical neurons were used. Error bars indicates mean ± SD from at least 20 cells/neurites from 3 independent experiments (n ≥ 20). *P < 0.05, **P < 0.01, ***P < 0.001, n.s., not significant; one-way ANOVA test. Scale bar, 10 μm

SIRT family proteins may regulate mitochondrial homeostasis including mitophagy [36]. To elucidate the mechanism of neurite protection and mitophagy activation of SAL, we measured the expression levels of mitochondrial SIRT3-5 in SH-SY5Y cells with and without SAL treatment. Interestingly, quantitative PCR (qPCR) assays indicated that SAL upregulated both SIRT3 and SIRT4, but not SIRT5 (Fig. 3A). Given that SIRT3 is the most thoroughly studied mitochondrial SIRT and its higher expression induced by SAL, our next study mainly focuses on SIRT3. Consistently, immunoblotting also showed a dose-dependent upregulation of SIRT3 protein levels upon SAL treatment, but with no change on the levels of APP and BACE1 (Fig. 3B). Although we may not entirely exclude other possible targets of SAL involved in this process, our data strongly suggest that SIRT3 is a critical molecule responsible for SAL-mediated neurite protection.

To answer that whether SIRT3 is essential for SAL-activated mitophagy, we generated two SH-SY5Y cell lines, in which, one stably expressed shRNA against SIRT3 [SIRT3 knockdown (KD)] and another overexpressed SIRT3 (SIRT3 OE) (Additional file 1: Fig. S5B, D and E). The data showed that the colocalization of LC3 and mitochondria was not increased after SAL treatment in SIRT3 KD cells, but upregulated in SIRT3 OE cells even without SAL treatment (Additional file 1: Fig. S4). Notably, not like in naïve cells, Aβ-induced mitophagy in SIRT3 KD cells was not further augmented by SAL either in somata, or in neurites (Fig. 3C–E), suggesting the exclusive function of SIRT3 needed. To further confirm the SIRT3 function, we cultured primary neurons from *Sirt3*^*floxp*^ mice and transduced them with AAV particles encoding MitoGFP and a Cre recombinase to obtain SIRT3 knockout (KO) cells in which mitochondria were simultaneously labeled with GFP (Additional file 1: Fig. S5A). IF images showed a similar effect of SAL in SIRT3 KO primary neurons as in SIRT3 KD cells (Fig. 3F–H).

Taken together, our data demonstrate that SIRT3 expression is indispensable for SAL-mediated activation of mitophagy.

### SIRT3 is required for SAL-induced neurite and mitochondrial protection

**Fig. 4:**
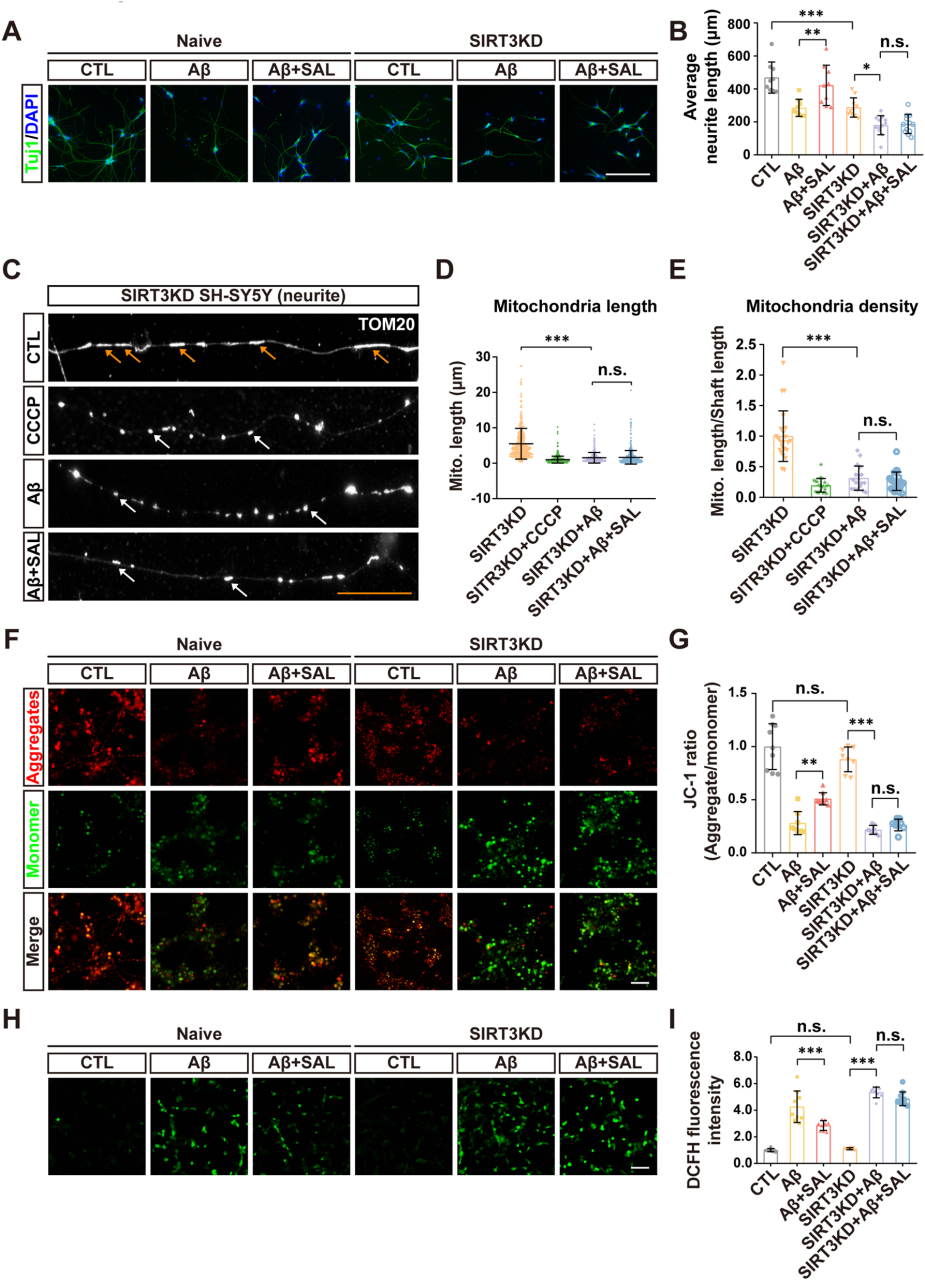
SIRT3 KD inhibits SAL-mediated neurite and mitochondrial protection. **A** IF of Tuj1 (green) shows neurite morphology in Naïve and SIRT3KD SH-SY5Y cells with indicated treatment. Nuclei counterstained with DAPI. **B** Quantification of neurite length of **A**. **C** IF of TOM20 shows mitochondrial morphology in neurites of SIRT3KD SH-SY5Y cells with indicated treatment. Orange arrows, healthy mitochondria; white arrows, damaged mitochondria. Quantitation of images in **C** shows the length of mitochondrial segments (D) and mitochondrial density (**E**). **F** Fluorescence images show the intensity of aggregates (red) and monomers (green) of JC-1 staining in naïve and SIRT3KD cells with indicated treatment. **G** Quantification of JC-1 ratio of intensity in **F**. **H** Images show the fluorescence intensity of DCFH (ROS indicator) in cells with indicated treatment. **I** Quantitation of fluorescence intensity in **H**. Error bars indicate mean ± SD. *P < 0.05, **P < 0.01, ***P < 0.001; n.s., not significant; one-way ANOVA test. White scale bar, 50 μm; orange scale bar, 10 μm

To verify the possible function of SIRT3 on neurite protection, we used both naïve SH-SY5Y and SIRT3 KD cell line to differentiate neurites and then conducted Aβ-induced neurite injury assays. Truly, SAL exhibited a powerful neurite protection in naïve SH-SY5Y cells when treated with Aβ. In SIRT3 KD cells, Aβ triggered a more severer neurite damage, however the SAL protection was completely abolished (Fig. 4A, B). Our data thus demonstrate the essentiality of SIRT3 for SAL-mediated neurite preservation.

Besides the activity of SIRT3 in mitophagy (Fig. 3), previous studies have also noted its function in the maintenance of mitochondrial homeostasis [37, 38]. We thereby investigated the potential capacity of SIRT3 in the homeostasis regulation of mitochondria by analyzing their morphology, membrane potential, and ROS production. Using IF of TOM20 (a mitochondrial marker), we detected the mitochondrial morphology in SIRT3 KD cells and found that mitochondrial segments in neurite were severely fragmented either by CCCP or Aβ treatment. Clearly, in SIRT3 KD cells, SAL did not show any protection on Aβ-induced mitochondrial damage, indicating by no restored mitochondrial length and density (Fig. 4C–E). The JC-1 assays also showed that the mitochondrial membrane potential (MMP) was impaired upon Aβ stimulation. Similarly, SAL treatment largely reduced the Aβ-induced MMP dysfunction in naïve cells, but its protection was similarly diminished in SIRT3 KD cells (Fig. 4F, G). We further measured the cellular ROS levels using a fluorescence probe DCFH. In accordance, Aβ-induced ROS upregulation was inhibited by SAL, only in naïve cells, not in SIRT3 KD cells (Fig. 4H, I).

Thus, our findings refer that SIRT3 is also requisite for SAL-mediated maintenance of mitochondrial homeostasis in neurite protection.

### SIRT3 is essential for SAL-mediated cognitive restoration of AD mice

**Fig. 5:**
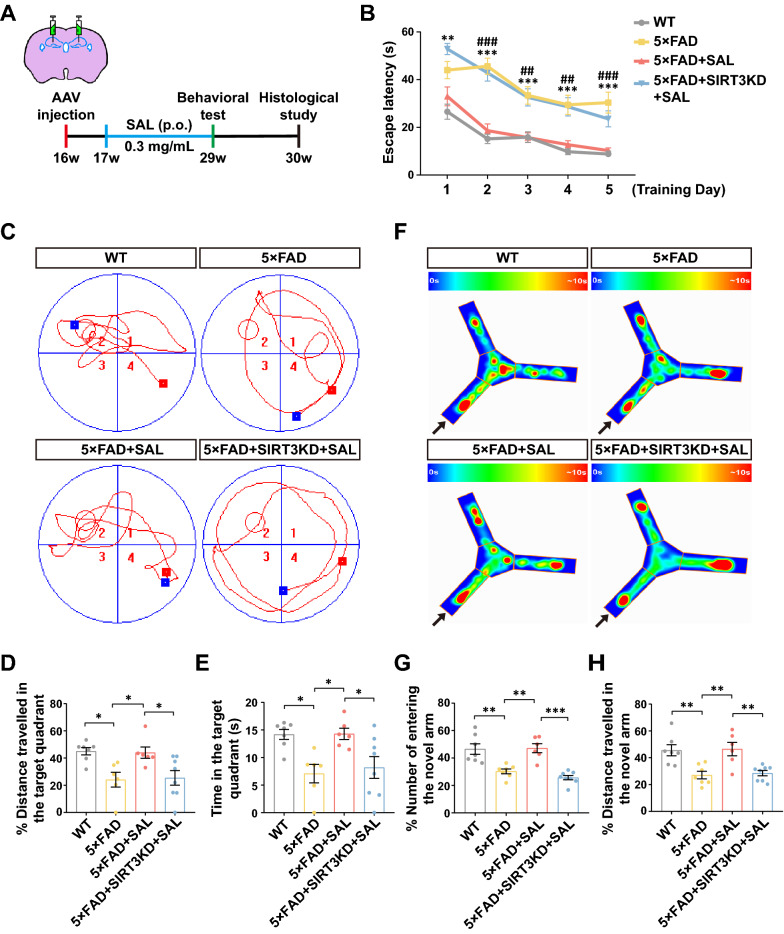
SAL-mediated cognitive protection is dependent on SIRT3. **A** A schematic overview shows the workflow of our animal study. **B** Quantitation shows the escape latency of mice from indicated groups from day 1 to day 5 during training. Significant differences are found in 5×FAD group compared to WT (**P < 0.01, ***P < 0.001) or 5×FAD+SAL group (^##^*P* < 0.01, ^###^*P* < 0.01). **C** Representative trajectories of mice from each group in MWM tests at day 6. Red square, starting point; blue square, ending point; platform (red circle) located in quadrant 2. Quantitation shows the traveled distance (**D**) and time spent (**E**) in the platform located quadrant. **F** Representative heatmap images show the visit frequency of mice in Y-maze. Arrow, the novel arm. Quantification shows the times of entry (**G**) and traveled distance (**H**) in the novel arm. All data are represented as mean ± SEM. *P < 0.05, **P < 0.01, ***P < 0.001, n.s., not significant; n ≥ 6; one-way ANOVA

We next extended our study into a 5×FAD mouse model of AD and a workflow diagram was described to assess the SAL efficacy and SIRT3 function (Fig. 5A). Morris water maze (MWM) test showed that 5×FAD had a clear deficiency of spatial memory in contrast to wild type (WT) mice. Indeed, oral administration of SAL sharply improved the learning and memory (Fig. 5B–E), which was in line with previous data from other AD animal models, in which SAL reduced cognitive impairment as well [39, 40]. We used an AAV injection approach for SIRT3 KD and successfully transduced the cells in the whole hippocampus (Additional file 1: Fig. S5A and S6A). As expected, SIRT3 KD in WT did not change the mouse behavior (Additional file 1: Fig. S6B–D). Strikingly, SIRT3 KD in hippocampi of 5×FAD, nearly completely abolished the protective efficacy of SAL (Fig. 5B–E). Moreover, we used Y-maze test to measure spatial reference memory. In accordance with the MWM test, it revealed that SAL strongly improved cognitive function in 5×FAD mice. Again, SIRT3 KD blocked the protective effect of SAL (Fig. 5F–H). Overall, these data imply that SAL can ameliorate cognitive decline in AD mice, which is dependent on SIRT3 expression.

### SAL mediates a SIRT3-dependent reduction of Aβ pathology and neurite damage in AD mice

**Fig. 6:**
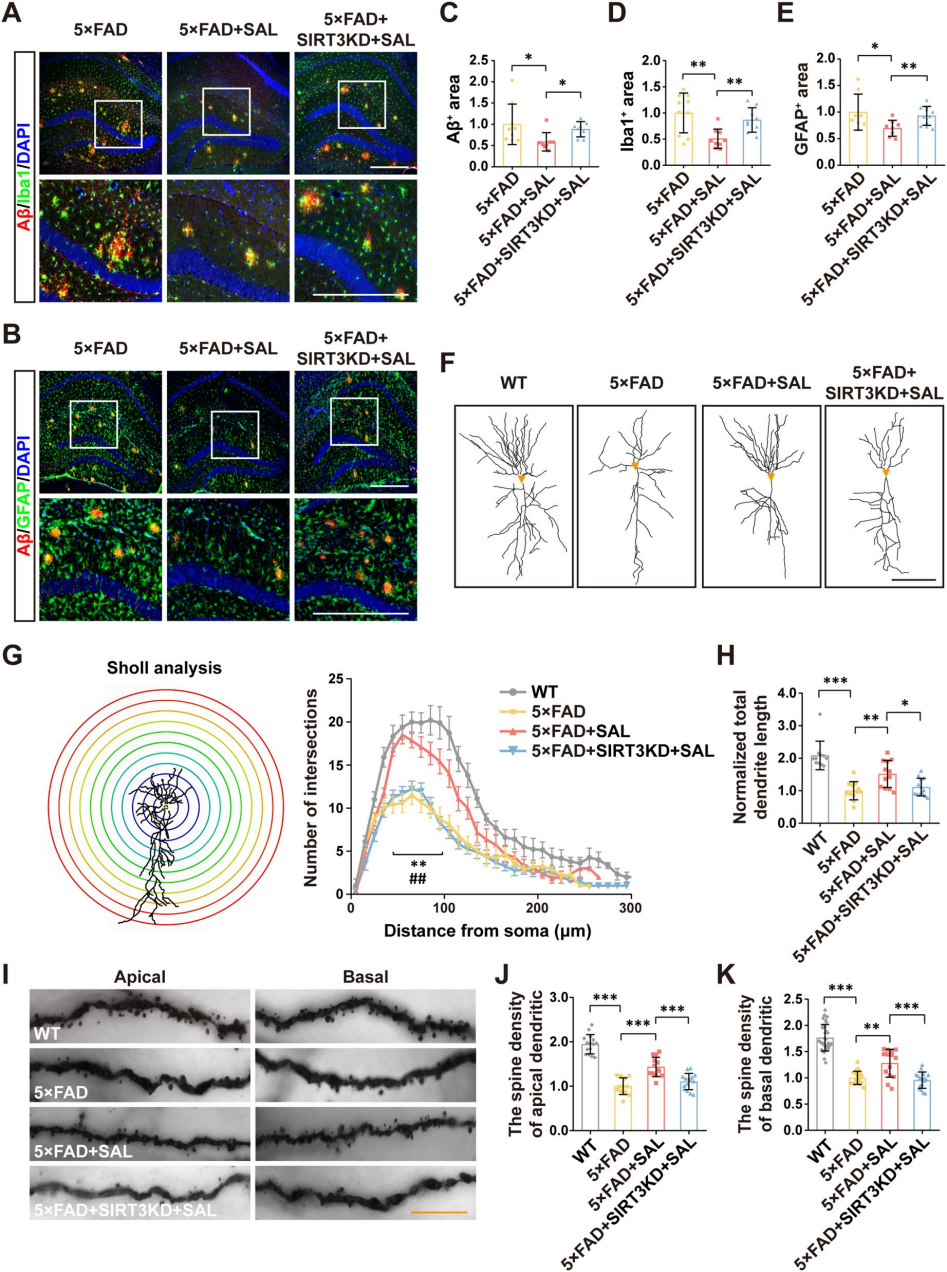
SAL alleviates Aβ pathology and restores neurite morphology in AD mice via SIRT3 action. **A** IHC staining of Aβ (red) and Iba1 (green) in hippocampi of 5×FAD mice. Nuclei counterstained with DAPI (blue). White rectangles indicate amplified images at lower panels. **B** As in **A**, except Aβ (red) and GFAP (green) are stained. Quantification shows the Aβ^+^ areas (**C**), Iba1^+^ areas (**D**) and GFAP^+^ areas (**E**). **F** The tracing images show the morphology of Golgi-stained CA1 pyramidal neurons in mice with indicated treatments. Orange triangle indicates the soma. **G** Sholl analysis of images collected in **F**. Significant differences are found in 50 μm to 100 μm (X-axis) in 5×FAD+SAL group compared to 5×FAD (**P < 0.01) or 5×FAD+SIRT3KD+SAL group (^##^*P* < 0.01). **H** Quantification of total dendrite length of cells in **F**. **I** Golgi-stained images show the morphology of apical and basal dendrites of CA1 pyramidal neurons. Quantification shows the spine density on apical dendrites (**J**) and basal dendrites (**K**). Data mean ± SD from at least 12 slices of 6 mice. *P < 0.05, **P < 0.01, ***P < 0.001; one-way ANOVA. White scale bar, 500 μm; black scale bar, 50 μm; orange scale bar, 5 μm

To elucidate whether SAL can mitigate Aβ pathology, a main cause for cognitive defect in 5×FAD, we examined the intensity of Aβ plaque, microgliosis and astrogliosis in hippocampi of 5×FAD mice. In fact, SAL greatly reduced the area of Aβ plaque (Aβ^+^ IHC) by 41.9%, microgliosis (Iba1^+^ IHC) by 49.3%, and astrogliosis (GFAP^+^ IHC) by 31.4%, respectively. We further analyzed the total number of Aβ plaques and the number of Aβ plaques with different sizes (diameter < 20 μm, 20–40 μm and > 40 μm). Data showed a SAL-mediated reduction of Aβ load, especially on medium and larger size of Aβ plaques (Additional file 1: Fig. S7). Consistent with the behavioral tests, SIRT3 KD inhibited the SAL-mediated reduction of these pathological features (Fig. 6A–E and Additional file 1: Fig. S7).

Next, we aimed to test whether SAL could alleviate neurite damage in brains of 5×FAD mice, like it does in cell cultures. Although knowing the difficulty of the technology, we tried to use IHC and detect the LAMP1 level as a marker for dystrophic neurites. Data indeed showed that SAL reduced the levels of LAMP1 nearby Aβ plaques, while SIRT3KD partially inhibited the SAL function (Additional file 1: Fig. S8). Next, we used Golgi silver staining to observe and trace neurite morphology of CA1 pyramidal neurons. The drawings in Fig. 6F displayed that the morphology of CA1 neurons in 5×FAD was simplified in contrast to WT, while SAL treatment evidently rescued this phenotype. Likewise, SIRT3 KD minimized the SAL potency on neurite protection (Fig. 6F). The sholl analysis, as established previously [41], allowed the quantification of branching and length of the dendrites. As expected, 5×FAD had fewer dendritic intersections than WT at a distance from soma around 50–100 μm, and SAL markedly increased dendritic intersections, across the same region (*P* < 0.01). Of note, SIRT3 KD did significantly blocked SAL efficacy (*P* < 0.01) (Fig. 6G). Conformably, the quantitation of total dendritic length showed a promotive effect of SAL, that was still dependent on SIRT3 expression (Fig. 6H). Using the same Golgi staining, we further studied the spines locating at both apical and basal dendrites. In keeping with the results of dendritic arborization (Fig. 6F–H), SAL showed a favorable function on spine density, in a similar fashion, contingent on the expression of SIRT3 (Fig. 6I–K).

Collectively, these results support the notion that SAL alleviates Aβ pathology and dendritic impairment in brains of AD mice and its efficacy is dependent on SIRT3 expression.

### SAL upregulates SIRT3 transcription by directly inhibiting NRF2-KEAP1 complex formation

**Fig. 7:**
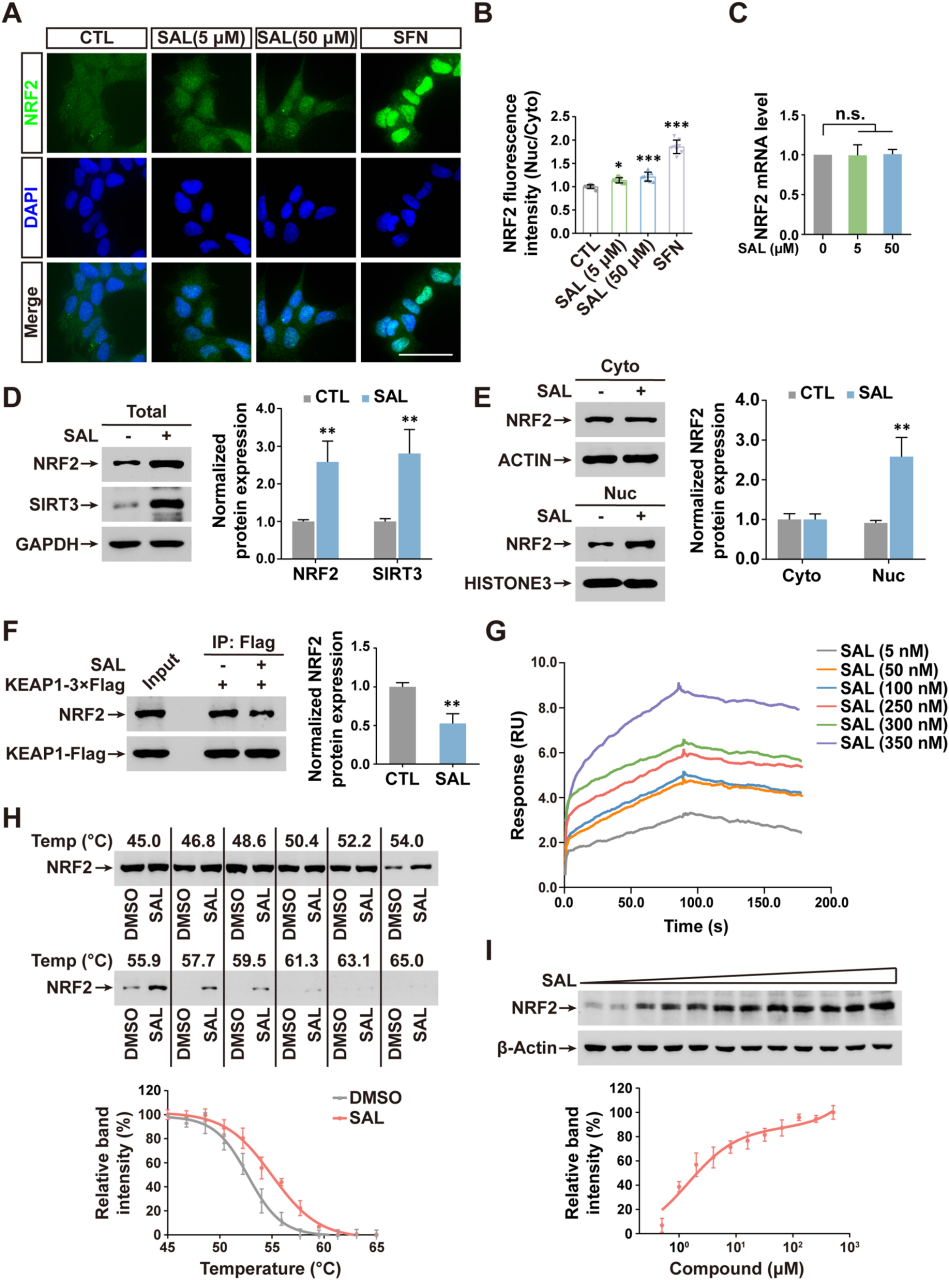
SAL directly inhibits NRF2-KEAP1 complex and facilitates NRF2-induced SIRT3 transcription. **A** IF of NRF2 (green) and nuclei (blue) in SH-SY5Y cells. Nuclei counterstained with DAPI. Scale bar, 50 μm. **B** Quantification shows the nucleus-to-cytoplasm ratio of NRF2 levels. **C** qPCR shows the NRF2 mRNA levels in SH-SY5Y cells treated with indicated dose of SAL. **D** Immunoblotting images (left) and quantification of band intensity (right) show the protein levels of NRF2 and SIRT3 in SH-SY5Y cells with or without SAL treatment. **E** As in **D**, except cytosolic (Cyto) and nuclear (Nuc) protein were isolated and detected. **F** Immunoprecipitation (IP) shows the protein levels of NRF2 and KEAP1-Flag in SH-SY5Y cells with indicated treatment. An anti-Flag antibody was used for IP. 5% total protein used as input. **G** Surface plasmon resonance (SPR) analyses show the SAL-NRF2 interaction behavior. Quantitation shows the sensogram of each indicated titration of SAL, as expressed in RU (response units-Y axis) along time (X-axis). **H** Cellular thermal shift assays (CETSA) show the binding capacity of SAL-NRF2 interaction. Immunoblotting shows NRF2 levels and below graph shows the CETSA melting curve. **I** Immunoblotting shows the NRF2 levels in protein lysates at 55 °C. The below graph shows the ITDR-CETSA curve. Error bars indicate mean ± SD from at least 3 independent experiments. *P < 0.05, **P < 0.01, ***P < 0.001, n.s., not significant; one-way ANOVA

Considering that the transcription factor NRF2 can regulate SIRT3 expression in some systems including aging process [42, 43], we thus hypothesized that SAL may regulate NFR2 to mediate SIRT3 transcription. To test our hypothesis, we first used immunofluorescence to check the NRF2 localization in SH-SY5Y cells incubated with low and high concentration of SAL, or potent NRF2 activator sulforaphane (SFN). As expected, SFN strongly increased nuclear levels of NRF2. Interestingly, either 5 μM or 50 μM SAL induced an obvious translocation of NRF2 from cytosol to nuclei, although with a milder potency compared to SFN (Fig. 7A, B). In addition, immunoblotting showed that SAL treatment upregulated the protein levels of NRF2 as well as SIRT3 (Fig. 7D). However, the mRNA levels of NRF2 did not change (Fig. 7C). These data refer that the NRF2 upregulation was not due to its transcription. By detecting nuclear and cytosolic NRF2, we further confirmed that SAL promoted the nuclear translocation of NRF2 (Fig. 7E). Given that SIRT3 mRNA was induced by SAL (Fig. 3A), a possible mechanism of NRF2-mediated SIRT3 transcription was further tested in the next study.

The E3 ligase adaptor KEAP1 is a principle negative regulator for NRF2. Thereby, we tested whether SAL could disrupt the formation of NRF2-KEAP1 protein complex. In KEAP1 immunoprecipitation (IP), 50 μM SAL definitely reduced the interaction of NRF2 with KEAP1 (Fig. 7F). We also checked whether SAL directly bound to NRF2 by using a real-time surface plasmon resonance (SPR) technology. To obtain the pure NRF2 protein for SPR use, we expressed the recombinant human NRF2 in Ecoli and purified it, showing by the Coomassie staining (Additional file 1: Fig. S9). SPR data indicated that SAL truly bound to NRF2 with a strong affinity (Fig. 7G), demonstrating NRF2 as a direct target of SAL. The interaction of SAL and NRF2 was further confirmed by the cellular thermal shift assays and data showed that induced a much stronger stability of NRF2 protein (Fig. 7H, I).

Thus, our findings demonstrate that SAL directly binds to NRF2 and promotes its nuclear translocation by inhibiting attachment of the KEAP1 E3 ligase, ultimately resulting in an elevated transcription of SIRT3.

## Discussion

**Fig. 8:**
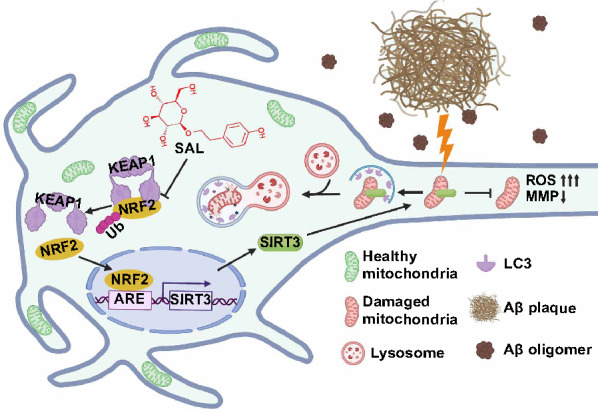
A graphic summary of the present study. A schematic drawing shows the working model of SAL-mediated neurite and mitochondrial protection in AD. Our findings suggest that SAL-mediated neurite and mitochondrial protection is dependent on SIRT3 expression. An elucidated mechanism of action is shown: SAL directly binds to transcription factor NRF2 and prevents its degradation by inhibiting the attachment of E3 ligase KEAP1, then promotes nuclear translocation of NRF2, and eventually favors SIRT3 transcription

Salidroside (SAL), a natural phenylpropanoid glycoside, is the main bioactive compound extracted from *Rhodiola* plants. A number of studies have shown that SAL has neuroprotective activities in multiple neurological diseases including stroke, mood disorder, Parkinson’s disease (PD) and AD [22]. Of note, being free of side effect makes SAL attractive as a drug candidate. The mechanisms of SAL protection possibly include the regulation of oxidative stress, inflammation, apoptosis and even neural regeneration [22]. However, the definite target molecules of SAL remain so far unknown, which largely limits its pharmacological study and clinical application. In the present study, we identified NRF2 as a direct binding target of SAL in neuronal cells (Fig. 7). As for the mechanism of action, we found that SAL disrupted NRF2-KEAP1 interaction, promoted NRF2 translocating into nuclei, and facilitated SIRT3 expression for mitochondrial and neurite protection (Fig. 8). These findings thus provide new insights into SAL target identification and its pharmacological mechanism of action in AD, and possibly, a general working model in other NDs.

It’s long been thought that the formation of dystrophic neurites is a vital event in AD pathogenesis [4]. The damaged neurites are mainly caused by toxic Aβ oligomers released from the local aggregated senile plaques. Given this pathological feature in AD, many studies mimicking the Aβ toxicity have been done by using various in vitro neuronal culture systems [44, 45]. By culturing both differentiated SH-SY5Y cells and primary neurons, we found for the first time that SAL attenuated Aβ-induced neurite damage (Fig. 1). In accordance with previous studies in other AD models [39, 40] and our in vitro data (Fig. 1), oral administration of SAL in 5×FAD mice also largely rescued cognitive defect, Aβ pathologies and neurite morphology (Figs. 5 and 6). But how can SAL reduce these AD pathological features? The answers may include protective effects of SAL on neurons shown here, or its possible function on other glia cells such as microglia and astrocytes, because SAL was also reported to modulate microglia activity in spinal cord injury [46], and astrocyte reaction in cerebral ischemia [47]. Thus, we cannot entirely exclude that the SAL-mediated neuroprotection in AD mice is due to its indirect effect on glia cells. It will be certainly interesting to investigate the gene expression profile and cell morphology of microglia or astrocytes in AD mice, particularly upon SAL treatment.

The regulation of mitochondrial dynamics and homeostasis is crucial for either neurite outgrowth in development, or neurite injury upon pathogen stimulation [48–50]. We then sought to assess the mitochondrial behavior during the process of SAL-mediated neurite protection. The data showed that the mitochondrial morphology was abnormal when cells treated with Aβ oligomers or toxic CCCP, and SAL indeed restored the mitochondrial length and segment number in neurites (Fig. 1E–G). These findings thus refer the involvement of mitochondrial activity in SAL-dependent neurite protection. Recent findings strongly suggest that impaired mitophagy is likely critical for AD pathogenesis, and promotion of mitophagy may inhibit some AD pathologies [34, 51]. We thus speculated that mitophagy might be affected by SAL or Aβ treatment. By using mKeima assays, double labeling of LC3 and mitochondria, and immunoblotting of protein markers in mitophagy pathway, we found that either Aβ42 or SAL alone, upregulated the basal level of mitophagy, while additional SAL treatment, not blocked but enlarged Aβ42-induced mitophagy (Fig. 2 and Additional file 1: Fig. S4). These data suggest a possible reciprocal regulation: (1) Aβ42 induced mitochondrial damage, which directly recruited mitophagic machinery for clearance, lead to increased mitophagy index; (2) SAL pre-incubation already raised the basal level of mitophagy (Fig. 2A, B), which can be further augmented by Aβ stimulation. Our data thus support the notion that promotion of mitophagy may exert a protective function against AD pathogenesis. Data in Fig. 2C, D showed that SAL upregulated the protein levels of PINK1 in both cytosolic and mitochondrial fractions, suggesting an elevated PINK1 expression mediated by SAL, which is possibly regulated by gene transcription, protein translation or post-translational modulation. However, the detail mechanism of SAL-mediated promotion of mitophagy has not been fully addressed in this study and it deserves further investigation in the future. Besides mitophagy, we also found that SAL was able to improve the mitochondrial performance such as their morphology, MMP and ROS production (Fig. 4).

To elucidate the mechanism responsible for SAL protection against neurite and mitochondrial injury, we turned to investigate the sirtuin family of proteins (SIRTs), which were considered as important neuroprotective molecules in the nervous system [52]. Interestingly, SAL treatment upregulated the mRNA levels of nearly all the mitochondrial SIRTs, except SIRT5. Among them, we focused on SIRT3, as a most studied one in mitochondrial regulation. Strikingly, our data from SIRT3 KD, SIRT3 OE and SIRT3 KO cells all suggested that SIRT3 was absolutely required for SAL protection on neurites as well as mitochondria (Figs. 3 and 4). Our in vivo animal study confirmed that SAL had neuroprotective function against AD pathogenesis and its efficacy was also dependent on SIRT3 expression (Figs. 5 and 6). However, our limited animal study did not include the group of 5×FAD+SIRT3KD, which possibly could have severe cognitive defect than 5×FAD mice. Based on the fact that 5×FAD+SIRT3KD+SAL almost restored it a similar level to 5×FAD (Fig. 5), we cannot exclude that SAL may have some additional protective function independent of SIRT3. We next extended our study into the mechanism of SIRT3 upregulation, by focusing on NRF2, a transcription factor implicated in regulating SIRT3 expression [53]. Strikingly, our experiments demonstrated that SAL directly bound to NRF2 and inhibited the formation of KEAP1-NRF2 complex (Fig. 7E–H). The detachment of KEAP1 from NRF2 lead to increased nuclear translocation of NRF2, and ultimately favored SIRT3 expression.

In the future, it will be worth to address some critical questions including: (1) whether and how NRF2 regulates other molecules in SAL-mediated neuroprotection? and (2) whether and how SAL modulates the function of microglia or astrocytes in AD? However, in a larger conjecture, our findings support the notion that SAL serves for mitochondrial and neurite protection in AD, dependent on the NRF2/SIRT3 pathway (Fig. 8), which may exemplify a more general model in other NDs and also provide a potential AD therapy.

## Conclusions

In sum, we find that a potential anti-aging drug SAL, can ameliorate Aβ-mediated neurite and mitochondrial damage in both cell culture and the 5×FAD mice. Notably, SIRT3, as a vital regulator for mitochondrial homeostasis, is indispensable for SAL-mediated neuroprotection. We further identify NRF2 as the first direct target of SAL, and elucidate a precise mechanism of action by which, SAL targets NRF2/SIRT3 pathway and attenuates Aβ pathology, restores neurite morphology, and improves cognitive function in AD mice. Our studies thus reveal a novel mechanism of SAL neuroprotection in AD and may provide potential candidates for possible intervention of AD.

## Supplementary Information

**Additional file 1: Figure S1.** SAL promotes neurite elongation in SH-SY5Y cells. **Figure S2.** SAL represses CCCP-triggered neurite and mitochondrial damage. **Figure S3.** Simultaneous treatment of SAL inhibits Aβ-induced neurite damage. **Figure S4.** SIRT3 regulates SAL-mediated promotion of mitophagy. **Figure S5.** Schematic drawings of the plasmid constructures. **Figure S6.** SIRT3 KD in hippocampi by AAV injection does not affect cognitive performance in MWZ. **Figure S7.** SAL-mediated reduction of Aβ loads is SIRT3 dependent. **Figure S8.** SAL reduces neurite dystrophy surrounding Aβ plaques via SIRT3 action. **Figure S9.** Purification of NRF2 protein expressed in *E. coli*. **Table S1.** Antibodies used in this study. **Table S2.** PCR primers used in this study.

## Data Availability

Any additional data and materials are available from corresponding authors on reasonable request.

## Abbreviations

AAV: Adeno-associated virus
Aβ: Beta-amyloid
AD: Alzheimer’s disease
CETSA: Cellular thermal shift assay
DNs: Dystrophic neurites
HFIP: Hexafluoroisopropanol
IF: Immunofluorescence
IHC: Immunohistochemistry
IP: Immunoprecipitation
MMP: Mitochondrial membrane potential
MWM: Morris water maze
NAD^+^: Nicotinamide adenine dinucleotide
NDs: Neurodegenerative diseases
PD: Parkinson’s disease
PDL: Poly-d-lysine
qPCR: Quantitative PCR
ROS: Reactive oxygen species
SAL: Salidroside
SFN: Sulforaphane
SIRTs: Sirtuins
SOD2: Superoxide dismutase 2
SPR: Surface plasmon resonance
WT: Wild type

## References

[1] Perl DP (2010). Neuropathology of Alzheimer’s disease. Mt Sinai J Med.

[2] Vickers JC, Mitew S, Woodhouse A, Fernandez-Martos CM, Kirkcaldie MT, Canty AJ, McCormack GH, King AE (2016). Defining the earliest pathological changes of Alzheimer’s disease. Curr Alzheimer Res.

[3] DeTure MA, Dickson DW (2019). The neuropathological diagnosis of Alzheimer’s disease. Mol Neurodegener.

[4] Garcia-Marin V, Garcia-Lopez P, Freire M (2007). Cajal’s contributions to the study of Alzheimer’s disease. J Alzheimers Dis.

[5] Sadleir KR, Kandalepas PC, Buggia-Prevot V, Nicholson DA, Thinakaran G, Vassar R (2016). Presynaptic dystrophic neurites surrounding amyloid plaques are sites of microtubule disruption, BACE1 elevation, and increased Abeta generation in Alzheimer’s disease. Acta Neuropathol.

[6] Adalbert R, Nogradi A, Babetto E, Janeckova L, Walker SA, Kerschensteiner M, Misgeld T, Coleman MP (2009). Severely dystrophic axons at amyloid plaques remain continuous and connected to viable cell bodies. Brain.

[7] Carrillo-Mora P, Luna R, Colin-Barenque L (2014). Amyloid beta: multiple mechanisms of toxicity and only some protective effects?. Oxid Med Cell Longev.

[8] Reiss AB, Arain HA, Stecker MM, Siegart NM, Kasselman LJ (2018). Amyloid toxicity in Alzheimer’s disease. Rev Neurosci.

[9] Baranov SV, Baranova OV, Yablonska S, Suofu Y, Vazquez AL, Kozai TDY, Cui XT, Ferrando LM, Larkin TM, Tyurina YY (2019). Mitochondria modulate programmed neuritic retraction. Proc Natl Acad Sci USA.

[10] Chaturvedi RK, Beal MF (2008). Mitochondrial approaches for neuroprotection. Ann N Y Acad Sci.

[11] Lanzillotta C, Di Domenico F, Perluigi M, Butterfield DA (2019). Targeting mitochondria in Alzheimer disease: rationale and perspectives. CNS Drugs.

[12] Kupis W, Palyga J, Tomal E, Niewiadomska E (2016). The role of sirtuins in cellular homeostasis. J Physiol Biochem.

[13] Zhang Y, Anoopkumar-Dukie S, Arora D, Davey AK (2020). Review of the anti-inflammatory effect of SIRT1 and SIRT2 modulators on neurodegenerative diseases. Eur J Pharmacol.

[14] Ying Y, Lu C, Chen C, Liu Y, Liu YU, Ruan X, Yang Y (2021). SIRT3 regulates neuronal excitability of Alzheimer’s disease models in an oxidative stress-dependent manner. Neuromol Med.

[15] Ji Z, Liu GH, Qu J (2021). Mitochondrial sirtuins, metabolism, and aging. J Genet Genom.

[16] Liu Y, Cheng A, Li YJ, Yang Y, Kishimoto Y, Zhang S, Wang Y, Wan R, Raefsky SM, Lu D (1886). SIRT3 mediates hippocampal synaptic adaptations to intermittent fasting and ameliorates deficits in APP mutant mice. Nat Commun.

[17] Kerr JS, Adriaanse BA, Greig NH, Mattson MP, Cader MZ, Bohr VA, Fang EF (2017). Mitophagy and Alzheimer’s disease: cellular and molecular mechanisms. Trends Neurosci.

[18] Tran M, Reddy PH (2020). Defective autophagy and mitophagy in aging and Alzheimer’s disease. Front Neurosci.

[19] Cen X, Chen Y, Xu X, Wu R, He F, Zhao Q, Sun Q, Yi C, Wu J, Najafov A, Xia H (2020). Pharmacological targeting of MCL-1 promotes mitophagy and improves disease pathologies in an Alzheimer’s disease mouse model. Nat Commun.

[20] Xie C, Zhuang XX, Niu Z, Ai R, Lautrup S, Zheng S, Jiang Y, Han R, Gupta TS, Cao S (2022). Amelioration of Alzheimer’s disease pathology by mitophagy inducers identified via machine learning and a cross-species workflow. Nat Biomed Eng.

[21] Zhang J, Xiang H, Liu J, Chen Y, He RR, Liu B (2020). Mitochondrial Sirtuin 3: new emerging biological function and therapeutic target. Theranostics.

[22] Zhong Z, Han J, Zhang J, Xiao Q, Hu J, Chen L (2018). Pharmacological activities, mechanisms of action, and safety of salidroside in the central nervous system. Drug Des Dev Ther.

[23] Yan ZQ, Chen J, Xing GX, Huang JG, Hou XH, Zhang Y (2015). Salidroside prevents cognitive impairment induced by chronic cerebral hypoperfusion in rats. J Int Med Res.

[24] Li Q, Wang J, Li Y, Xu X (2018). Neuroprotective effects of salidroside administration in a mouse model of Alzheimer’s disease. Mol Med Rep.

[25] Shipley MM, Mangold CA, Szpara ML (2016). Differentiation of the SH-SY5Y human neuroblastoma cell line. J Vis Exp.

[26] Xu C, Wu J, Wu Y, Ren Z, Yao Y, Chen G, Fang EF, Noh JH, Liu YU, Wei L (2021). TNF-alpha-dependent neuronal necroptosis regulated in Alzheimer’s disease by coordination of RIPK1-p62 complex with autophagic UVRAG. Theranostics.

[27] Stine WB, Jungbauer L, Yu C, LaDu MJ (2011). Preparing synthetic Abeta in different aggregation states. Methods Mol Biol.

[28] Sima J, Yan Z, Chen Y, Lehrmann E, Zhang Y, Nagaraja R, Wang W, Wang Z, Schlessinger D (2018). Eda-activated RelB recruits an SWI/SNF (BAF) chromatin-remodeling complex and initiates gene transcription in skin appendage formation. Proc Natl Acad Sci USA.

[29] Kraeuter AK, Guest PC, Sarnyai Z (2019). The Y-maze for assessment of spatial working and reference memory in mice. Methods Mol Biol.

[30] Zaqout S, Kaindl AM (2016). Golgi-Cox staining step by step. Front Neuroanat.

[31] Jafari R, Almqvist H, Axelsson H, Ignatushchenko M, Lundback T, Nordlund P, Martinez Molina D (2014). The cellular thermal shift assay for evaluating drug target interactions in cells. Nat Protoc.

[32] Li R, Guo Y, Zhang Y, Zhang X, Zhu L, Yan T (2019). Salidroside ameliorates renal interstitial fibrosis by inhibiting the TLR4/NF-kappaB and MAPK signaling pathways. Int J Mol Sci.

[33] Lu H, Li Y, Zhang T, Liu M, Chi Y, Liu S, Shi Y (2017). Salidroside reduces high-glucose-induced podocyte apoptosis and oxidative stress via upregulating heme oxygenase-1 (HO-1) expression. Med Sci Monit.

[34] Fang EF, Hou Y, Palikaras K, Adriaanse BA, Kerr JS, Yang B, Lautrup S, Hasan-Olive MM, Caponio D, Dan X (2019). Mitophagy inhibits amyloid-beta and tau pathology and reverses cognitive deficits in models of Alzheimer’s disease. Nat Neurosci.

[35] Wang W, Zhao F, Ma X, Perry G, Zhu X (2020). Mitochondria dysfunction in the pathogenesis of Alzheimer’s disease: recent advances. Mol Neurodegener.

[36] Aventaggiato M, Vernucci E, Barreca F, Russo MA, Tafani M (2021). Sirtuins’ control of autophagy and mitophagy in cancer. Pharmacol Ther.

[37] Song Y, Li S, Geng W, Luo R, Liu W, Tu J, Wang K, Kang L, Yin H, Wu X (2018). Sirtuin 3-dependent mitochondrial redox homeostasis protects against AGEs-induced intervertebral disc degeneration. Redox Biol.

[38] Sack MN (2012). The role of SIRT3 in mitochondrial homeostasis and cardiac adaptation to hypertrophy and aging. J Mol Cell Cardiol.

[39] Gao J, He H, Jiang W, Chang X, Zhu L, Luo F, Zhou R, Ma C, Yan T (2015). Salidroside ameliorates cognitive impairment in a d-galactose-induced rat model of Alzheimer’s disease. Behav Brain Res.

[40] Wang H, Li Q, Sun S, Chen S (2020). Neuroprotective effects of salidroside in a mouse model of Alzheimer’s disease. Cell Mol Neurobiol.

[41] Beauquis J, Roig P, De Nicola AF, Saravia F (2010). Short-term environmental enrichment enhances adult neurogenesis, vascular network and dendritic complexity in the hippocampus of type 1 diabetic mice. PLoS ONE.

[42] Satterstrom FK, Swindell WR, Laurent G, Vyas S, Bulyk ML, Haigis MC (2015). Nuclear respiratory factor 2 induces SIRT3 expression. Aging Cell.

[43] Barroso E, Rodriguez-Rodriguez R, Zarei M, Pizarro-Degado J, Planavila A, Palomer X, Villarroya F, Vazquez-Carrera M (2020). SIRT3 deficiency exacerbates fatty liver by attenuating the HIF1alpha-LIPIN 1 pathway and increasing CD36 through Nrf2. Cell Commun Signal.

[44] Fontana IC, Zimmer AR, Rocha AS, Gosmann G, Souza DO, Lourenco MV, Ferreira ST, Zimmer ER (2020). Amyloid-beta oligomers in cellular models of Alzheimer’s disease. J Neurochem.

[45] Klein WL, Krafft GA, Finch CE (2001). Targeting small Abeta oligomers: the solution to an Alzheimer’s disease conundrum?. Trends Neurosci.

[46] Wang C, Wang Q, Lou Y, Xu J, Feng Z, Chen Y, Tang Q, Zheng G, Zhang Z, Wu Y (2018). Salidroside attenuates neuroinflammation and improves functional recovery after spinal cord injury through microglia polarization regulation. J Cell Mol Med.

[47] Dong C, Wen S, Zhao S, Sun S, Zhao S, Dong W, Han P, Chen Q, Gong T, Chen W (2021). Salidroside inhibits reactive astrogliosis and glial scar formation in late cerebral ischemia via the Akt/GSK-3beta pathway. Neurochem Res.

[48] Cheng A, Hou Y, Mattson MP (2010). Mitochondria and neuroplasticity. ASN Neuro.

[49] Chamberlain KA, Sheng ZH (2019). Mechanisms for the maintenance and regulation of axonal energy supply. J Neurosci Res.

[50] Rezaeian AH, Wei W, Inuzuka H (2022). The regulation of neuronal autophagy and cell survival by MCL1 in Alzheimer’s disease. Acta Mater Med.

[51] Hou X, Watzlawik JO, Cook C, Liu CC, Kang SS, Lin WL, DeTure M, Heckman MG, Diehl NN, Al-Shaikh FSH (2020). Mitophagy alterations in Alzheimer’s disease are associated with granulovacuolar degeneration and early tau pathology. Alzheimers Dement.

[52] Zhang F, Wang S, Gan L, Vosler PS, Gao Y, Zigmond MJ, Chen J (2011). Protective effects and mechanisms of sirtuins in the nervous system. Prog Neurobiol.

[53] Morris G, Walker AJ, Walder K, Berk M, Marx W, Carvalho AF, Maes M, Puri BK (2021). Increasing Nrf2 activity as a treatment approach in neuropsychiatry. Mol Neurobiol.

