# Supplementary material for: Salidroside reduces neuropathology in Alzheimer’s disease models by targeting NRF2/SIRT3 pathway
Source: Cell Biosci. 2022 Nov 4;12:180. doi: 10.1186/s13578-022-00918-z (PMC9636768; doi:10.1186/s13578-022-00918-z)
Supplement: Supplementary file 1 — Additional file 1: Figure S1. SAL promotes neurite elongation in SH-SY5Y cells. Figure S2. SAL represses CCCP-triggered neurite and mitochondrial damage. Figure S3. Simultaneous treatment of SAL inhibits Aβ-induced neurite damage. Figure S4. SIRT3 regulates SAL-mediated promotion of mitophagy. Figure S5. Schematic drawings of the plasmid constructures. Figure S6. SIRT3 KD in hippocampi by AAV injection does not affect cognitive performance in MWZ. Figure S7. SAL-mediated reduction of Aβ loads is SIRT3 dependent. Figure S8. SAL reduces neurite dystrophy surrounding Aβ plaques via SIRT3 action. Figure S9. Purification of NRF2 protein expressed in E. coli. Table S1. Antibodies used in this study. Table S2. PCR primers used in this study. [file 13578_2022_918_MOESM1_ESM.docx]

**Additional file 1**

**Salidroside reduces neuropathology in Alzheimer's disease models by targeting NRF2/SIRT3 pathway**

Yuyuan Yao^1#^, Zhichu Ren^1#^, Ruihan Yang^1^, Yilan Mei^1^, Yuying Dai^1^, Qian Cheng^1^, Chong Xu^1^, Xiaogang Xu^2^, Sanying Wang^2^, [Kyoung Mi Kim](https://pubmed.ncbi.nlm.nih.gov/?term=Kim+KM&cauthor_id=28877934)^3^, Ji Heon Noh^4^, Jian Zhu^5^, Ningwei Zhao^6^, Yong U. Liu^7^, Genxiang Mao^2*^ and Jian Sima^1*^

1. Laboratory of Aging Neuroscience and Neuropharmacology, School of Basic Medicine and Clinical Pharmacy, China Pharmaceutical University, Nanjing, 210009, China.
2. Zhejiang Provincial Key Lab of Geriatrics and Geriatrics, Institute of Zhejiang Province, Department of Geriatrics, Zhejiang Hospital, Hangzhou, 310030, China.
3. Department of Biological Sciences, Chungnam National University, Daejeon, 34134, Korea.
4. Department of Biochemistry, Chungnam National University, Daejeon, 34134, Korea.
5. Department of Psychology, Eastern Illinois University, Charleston, IL, 61920, USA.
6. China Exposomics Institute, 781 Cai Lun Road, Shanghai, 200120, China.
7. Laboratory for Neuroscience in Health and Disease, Guangzhou First People's Hospital, South China University of Technology, Guangzhou, 510180, China.

^#^ These authors contributed equally to this work.

* To whom correspondence should be addressed.

Jian Sima, Email: simajian@cpu.edu.cn;

Genxiang Mao, Email: maogenxiang@163.com


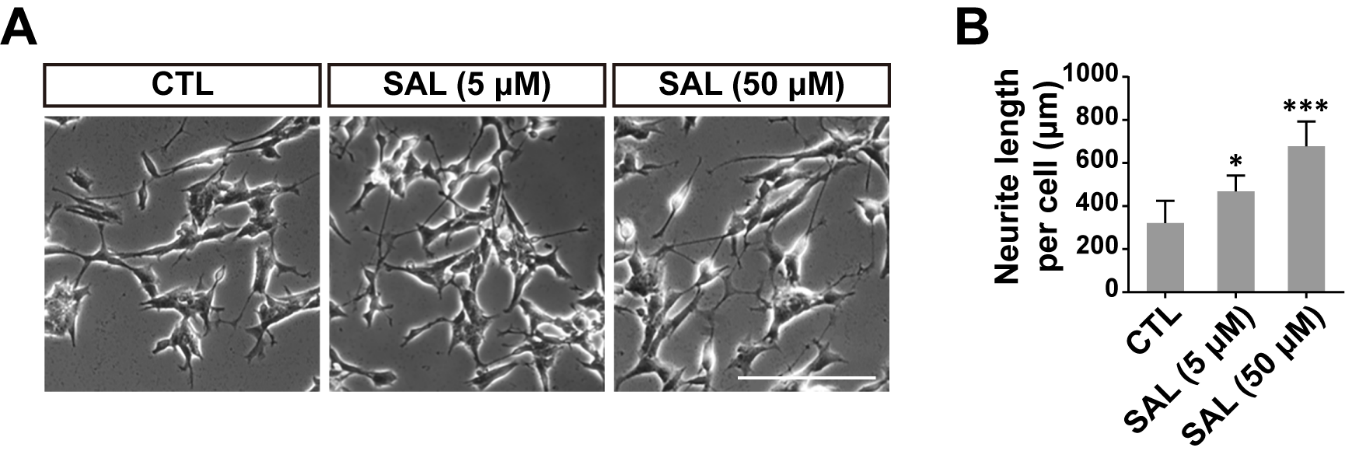


**Figure S1. SAL promotes neurite elongation in SH-SY5Y cells**

(A) Brightfield images show the morphology of differentiated SH-SY5Y cells with SAL treatment with indicated concentration. Scale bar, 50 μm. (B) Quantification of neurite length of (A). The average neurite length without additional treatment was regarded as control (CTL). Error bars indicate the mean ± SD. **P*<0.05, ****P*<0.001; one-way ANOVA test**.**


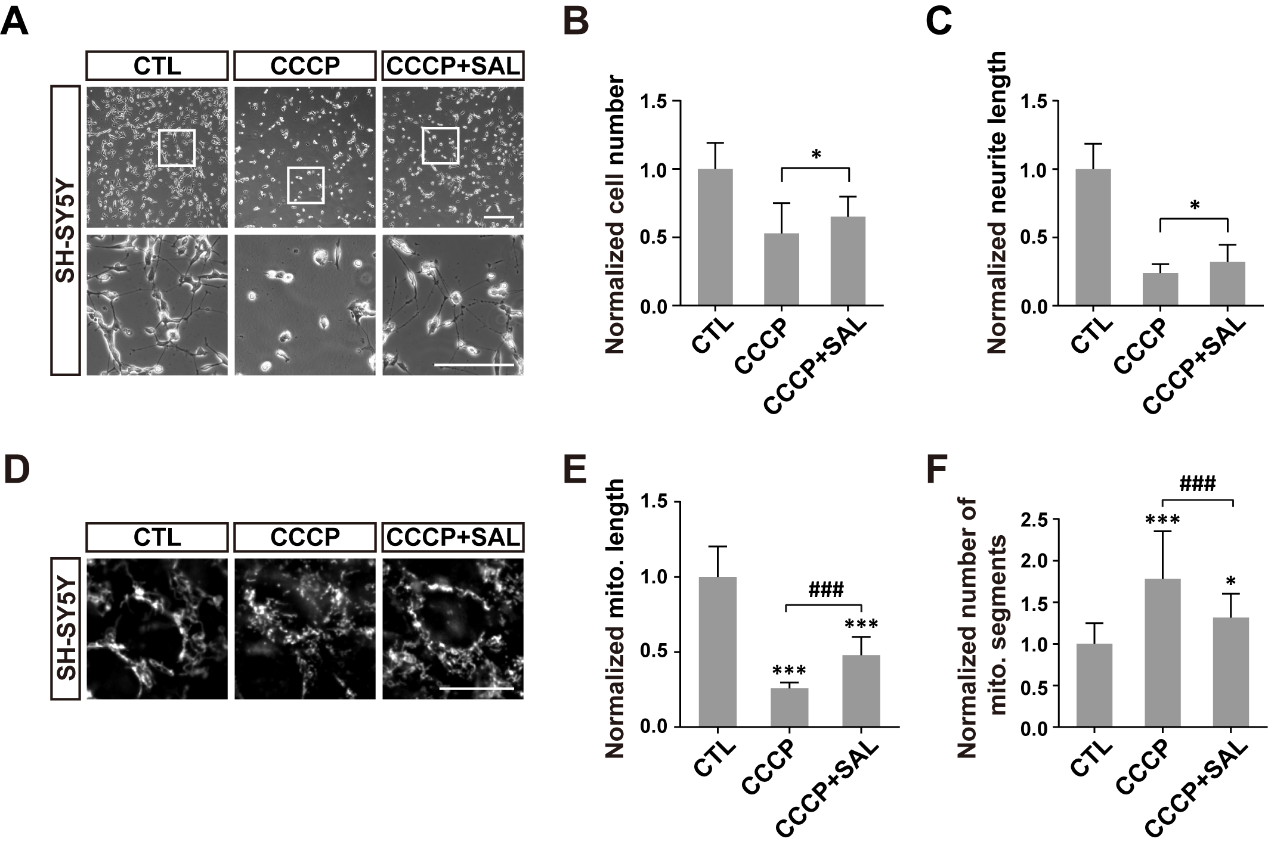


**Figure S2. SAL represses CCCP-triggered neurite and mitochondrial damage**

(A) Bright-field images show the morphology of differentiated SH-SY5Y cells with indicated treatment of vehicle (CTL), CCCP or CCCP combined with SAL. Scale bar, 50 μm. Quantitation shows the cell numbers (B) and neurite length (C) in each condition. (D) IF of TOM20 shows the mitochondrial morphology in SH-SY5Y cells with indicated treatment. Scale bar, 10 μm. Quantitative histograms show the length (E) and number (F) of mitochondrial segments. All error bars indicate mean ± SD. **P*<0.05, ****P*<0.001, ^###^*P*<0.001; n≥20; one-way ANOVA test.


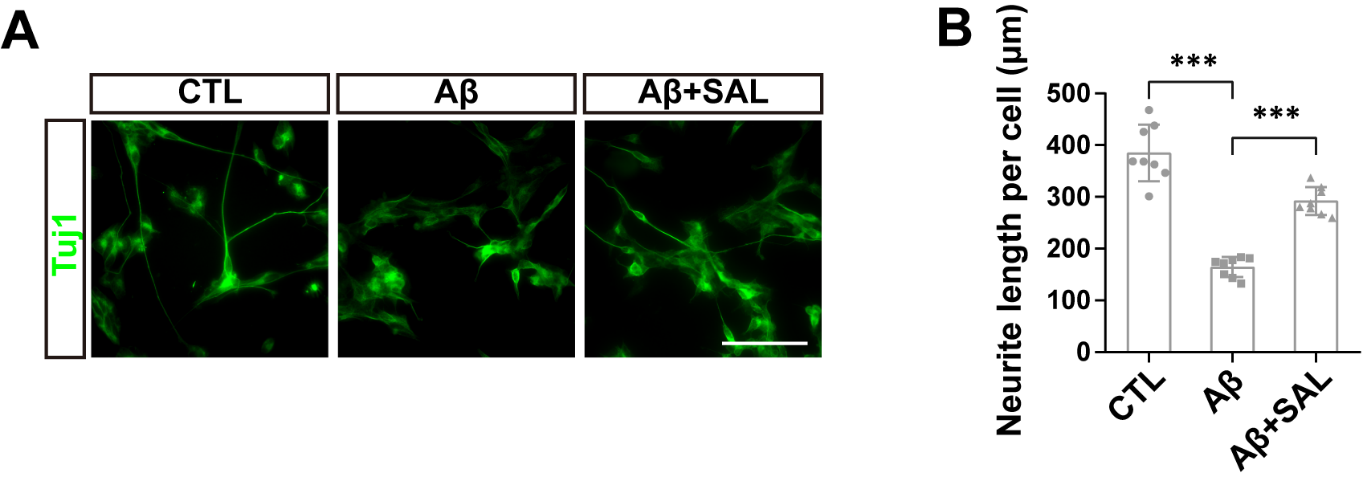


**Figure S3. Simultaneous treatment of SAL inhibits Aβ-induced neurite damage**

(A) IF of neuronal marker Tuj1 (green) in differentiated SH-SY5Y cells, treated with Aβ42 oligomers with or without 50 μM SAL for 24 h. Scale bar, 50 μm. (B) Quantification of neurite length of (A). The average neurite length without additional treatment was regarded as control (CTL). Error bars indicate the mean ± SD. ****P*<0.001; one-way ANOVA test**.**


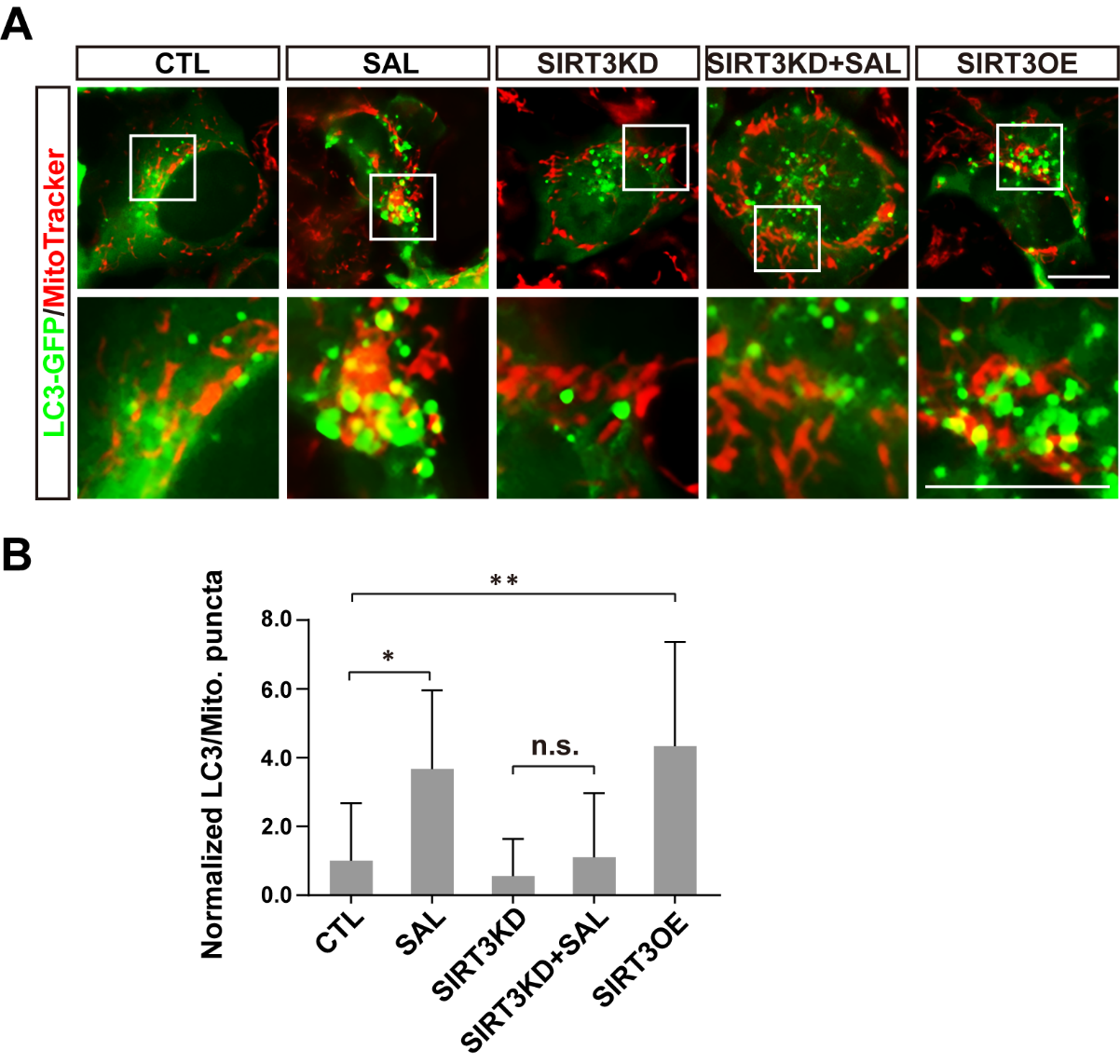


**Figure S4. SIRT3 regulates SAL-mediated promotion of mitophagy**

(A) Fluorescence images show the puncta labeled with a MitoTracker probe (red) and LC3-GFP (green) in naïve, SIRT3KD, and SIRT3OE SH-SY5Y cells with or without additional SAL treatment. Scale bar, 5 μm. Quantitation of co-localization of MitoTracker and LC3 is shown in (B). Error bars indicate mean ± SD. **P*<0.05, ***P*<0.01, n.s., not significant; n≥20; one-way ANOVA test.


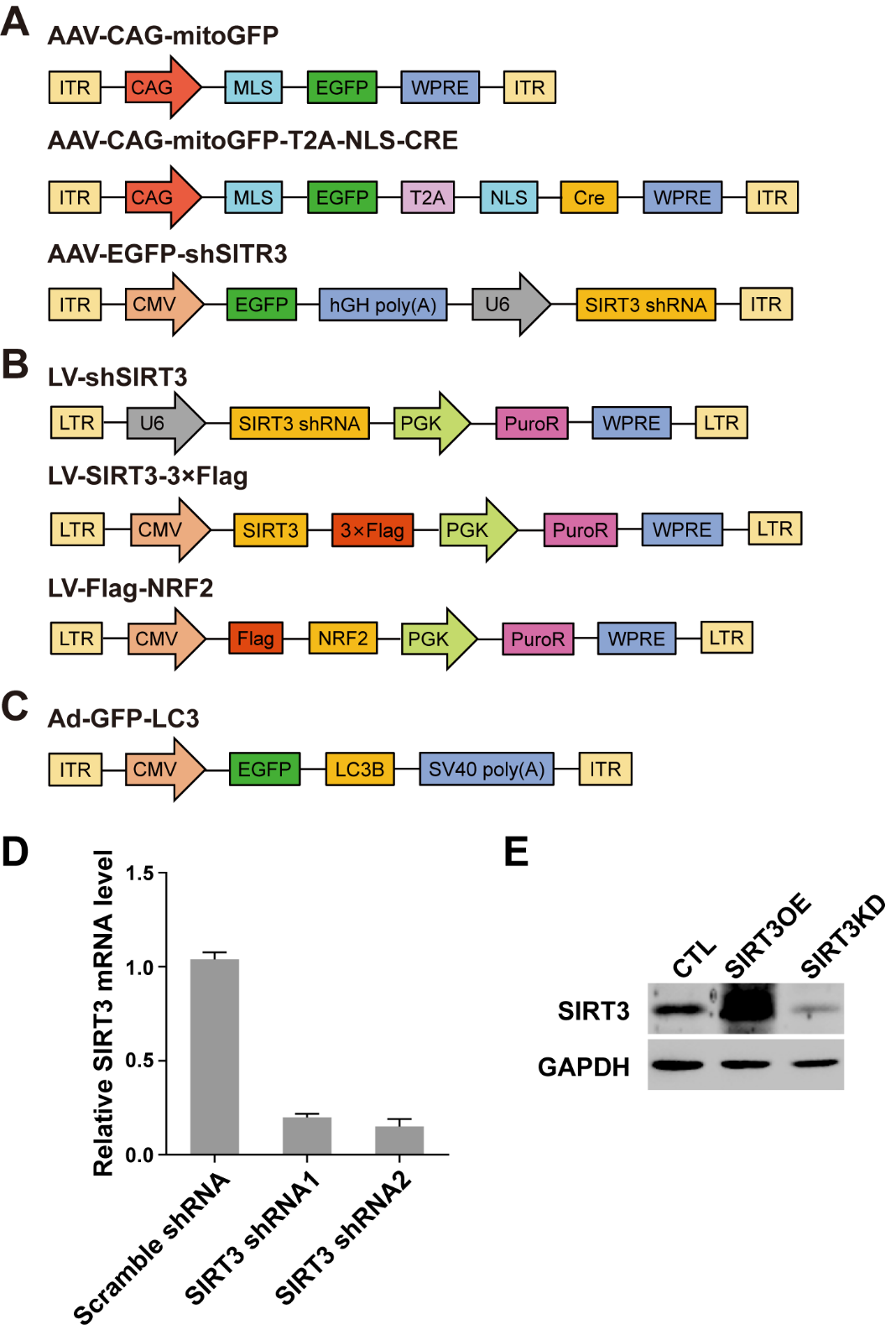


**Figure S5. Schematic drawings of the plasmid constructures**

Schematic structures of the adeno-associated viral (AAV) plasmids (A), HIV-1-based lentivial plasmids (B), and an adenoviral plasmid (C) used in this study. (D) qPCR shows the knockdown efficiency of SIRT3 shRNAs in SH-SY5Y cells. Error bars indicate mean ± SD. n≥3. (E) Immunoblotting shows the validation of SIRT3 overexpression (OE) and knockdown (KD) in SH-SY5Y cells.


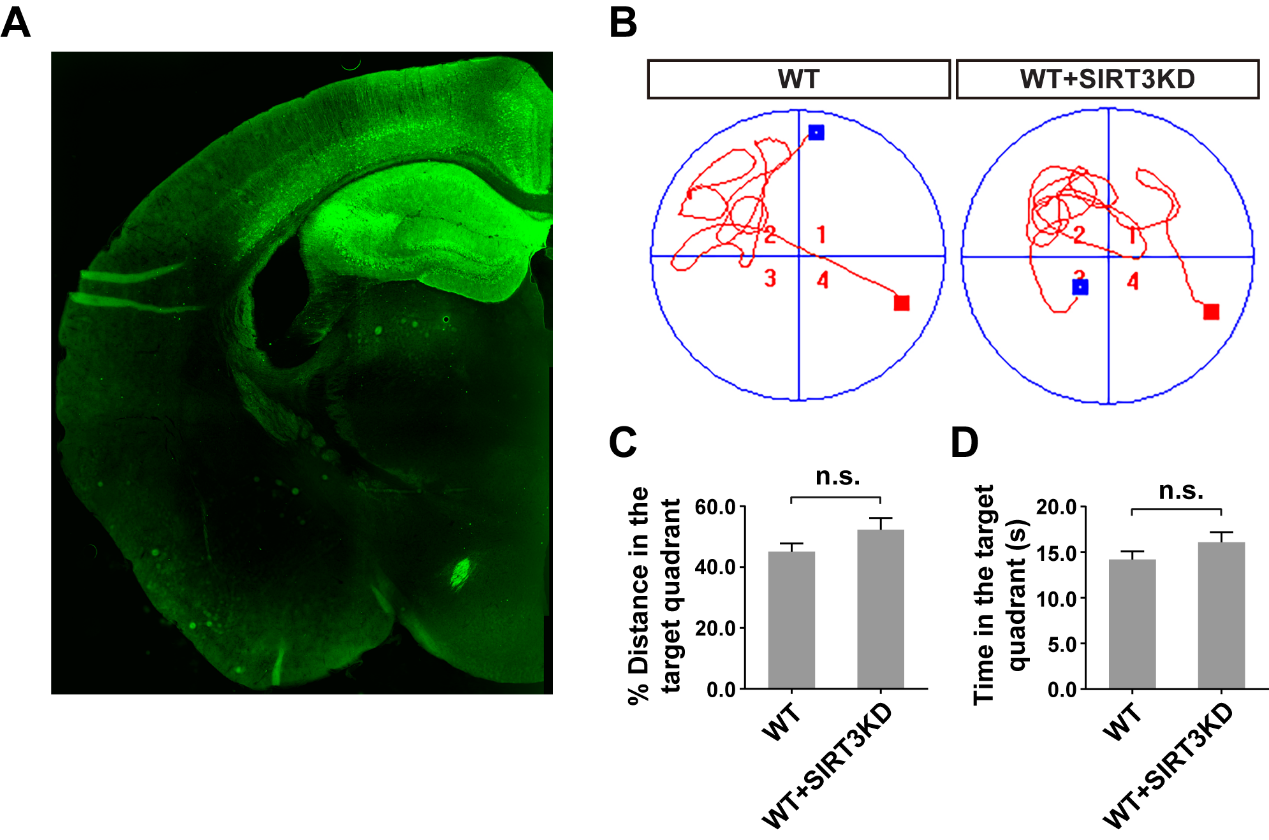


**Figure S6. SIRT3 KD in hippocampi by AAV injection does not affect cognitive performance in MWZ**

(A) A GFP image shows the efficiency of AAV transduction, indicating by the label of the whole hippocampus. (B) Representative trajectories of wild type (WT) mice with or without injection of AAV particles encoding shRNA against SIRT3. Quantitation of traveled distance (C) and time spent (D) in the platform located quadrant shows no significant difference between WT and SIRT3 KD groups. Error bars indicate mean ± SEM. n.s., not significant; n≥6; Student’s *t*-test.


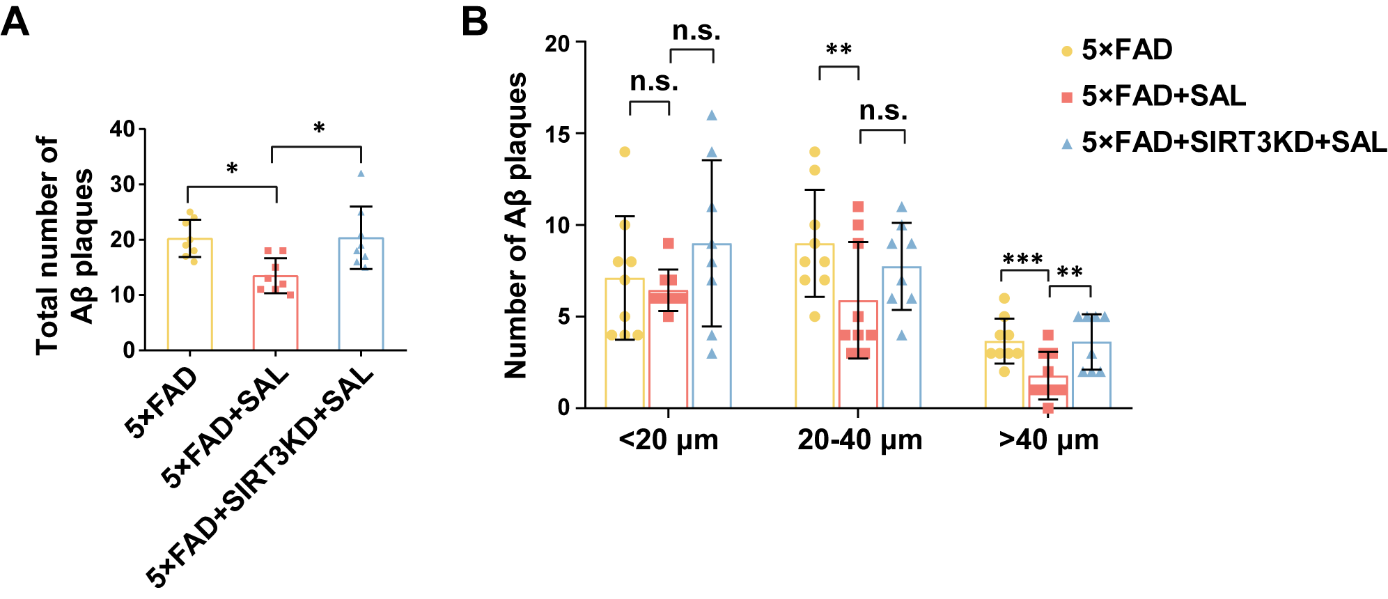


**Figure S7. SAL-mediated reduction of Aβ loads is SIRT3 dependent**

(A) Quantification shows total number of Aβ plaques per section (B) Quantification shows the average number of Aβ plaques with different sizes (diameter<20 μm, 20-40 μm and >40 μm) per section. Error bars indicate the mean ± SD. **P*<0.05, ***P*<0.01, ****P*<0.001; n.s., not significant; one-way ANOVA test**.**


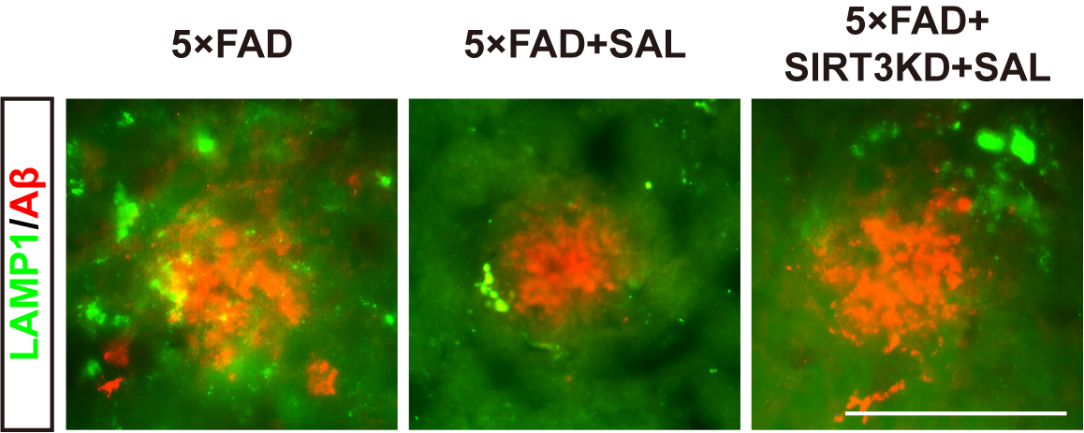


**Figure S8. SAL reduces neurite dystrophy surrounding Aβ plaques via SIRT3 action**

Representative IF images of LAMP1 (green) and Aβ (red) in hippocampi of 5×FAD mice with indicated treatments. Scale bar, 50 μm.


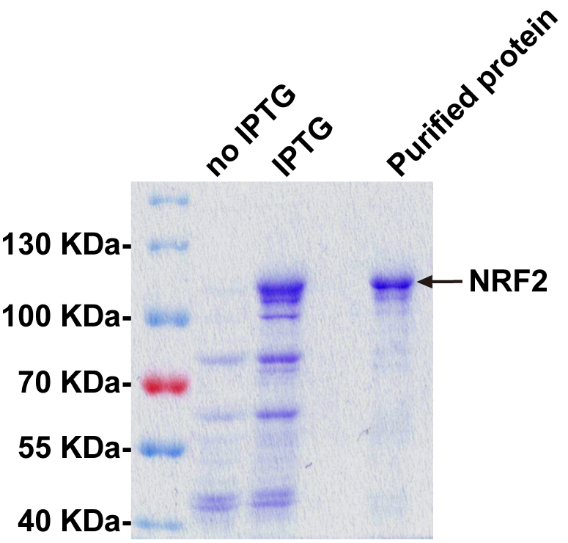


**Figure S9. Purification of NRF2 protein expressed in Ecoli**

A Coomassie stained polyacrylamide gel shows the amount and purity of NRF2 protein expressed and purified from Ecoli (see method). The arrow indicates the band of recombinant human NRF2. IPTG (isopropyl β-D-1-thiogalactopyranoside) used for induction of protein expression.

**Table S1. Antibodies used in this study**

| **Antibody** | **Vendor** | **Cat. #** |
| --- | --- | --- |
| Anti-PARKIN | Wanleibio | WL02512 |
| Anti-LC3 | [SigmaAldrich](https://www.sigmaaldrich.com/) | L7543 |
| Anti-PINK1 | ProteinTech | 23274-1-AP |
| Anti-GAPDH | ABclonal | AC033 |
| Anti-VDAC1/2 | ProteinTech | 10866-1-AP |
| Anti-Tuj1 | Cell Signaling Technology | 5568 |
| Anti-TOM20 | ProteinTech | 11802-1-AP |
| Anti-SIRT3 | ProteinTech | 10099-1-AP |
| Anti-APP | Proteintech | 25524-1-AP |
| Anti-BACE1 | Wanleibio | WL02795 |
| Anti-LAMP1 | Wanleibio | WL03761 |
| Anti-Aβ | Cell Signaling Technology | 2454 |
| Anti-GFAP | FUJIFILM Wako Pure Chemical | MO389 |
| Anti-Iba-1 | GeneTex | GTX100042 |
| Anti-DYKDDDDK tag | ProteinTech | 80010-1-RR |
| Anti-KEAP1 | Cell Signaling Technology | 4678 |
| Anti-NRF2 | Wanleibio | WL02135 |
| Anti-ACTIN | ABclonal | AC026 |
| Anti-HISTONE3 | Cell Signaling Technology | 4620 |
| Anti-Rabbit IgG, HRP-linked | Cell Signaling Technology | 7074 |
| Anti-Mouse IgG, HRP-linked | Cell Signaling Technology | 7076 |
| Anti-Rabbit IgG (H+L) Alexa Fluor(R)-488 | Thermo Fisher Scientific | A32731 |
| Anti-Rabbit IgG (H+L) Alexa Fluor(R)-546 | Thermo Fisher Scientific | A11003 |
| Anti-Rabbit IgG (H+L) Alexa Fluor(R)-568 | Thermo Fisher Scientific | A11011 |
| Anti-Rabbit IgG (H+L) Alexa Fluor(R)-350 | Thermo Fisher Scientific | A11045 |

**Table S2. PCR primers used in this study**

| **Primer name** | **DNA sequence (5’-3’)** |
| --- | --- |
| Human Sirt3-F | AGAAGAGATGCGGGACCTTG |
| Human Sirt3-R | GGTCCATCAAGCCTAGAGCAG |
| Human Sirt4-F | GGCAGGAATCTCCACCGAAT |
| Human Sirt4-R | GCACTCCGGACAAAATCACC |
| Human Sirt5-F | GGTGTTCCGACCTTCAGAGG |
| Human Sirt5-R | GTGGTAGAACTCCCACACCC |
| Mouse Sirt3-F | ATCCCGGACTTCAGATCCCC |
| Mouse Sirt3-R | CAACATGAAAAAGGGCTTGGG |
| Mouse Gapdh-F | AGGTCGGTGTGAACGGATTTG |
| Mouse Gapdh-R | TGTAGACCATGTAGTTGAGGTCA |
| Human Gapdh-F | GGACTCATGACCACAGTCCA |
| Human Gapdh-R | TCAGCTCAGGGATGACCTTG |
| Human Nrf2-F | TCAGCGACGGAAAGAGTATGA |
| Human Nrf2-R | CCACTGGTTTCTGACTGGATGT |
